# Cellular senescence impairs tendon extracellular matrix remodeling in response to mechanical unloading

**DOI:** 10.1111/acel.14278

**Published:** 2024-07-22

**Authors:** Emma J. Stowe, Madelyn R. Keller, Brianne K. Connizzo

**Affiliations:** ^1^ Department of Biomedical Engineering Boston University Boston Massachusetts USA

**Keywords:** aging, explant, extracellular matrix, remodeling, senescence, tendon

## Abstract

Musculoskeletal injuries, including tendinopathies, present a significant clinical burden for aging populations. While the biological drivers of age‐related declines in tendon function are poorly understood, it is well accepted that dysregulation of extracellular matrix (ECM) remodeling plays a role in chronic tendon degeneration. Senescent cells, which have been associated with multiple degenerative pathologies in musculoskeletal tissues, secrete a highly pro‐inflammatory senescence‐associated secretory phenotype (SASP) that has potential to promote ECM breakdown. However, the role of senescent cells in the dysregulation of tendon ECM homeostasis is largely unknown. To assess this directly, we developed an in vitro model of induced cellular senescence in murine tendon explants. This novel technique enables us to study the isolated interactions of senescent cells and their native ECM without interference from age‐related systemic changes. We document multiple biomarkers of cellular senescence in induced tendon explants including cell cycle arrest, apoptosis resistance, and sustained inflammatory responses. We then utilize this in vitro senescence model to compare the ECM remodeling response of young, naturally aged, and induced‐senescent tendons to an altered mechanical stimulus. We found that both senescence and aging independently led to alterations in ECM‐related gene expression, reductions in protein synthesis, and tissue compositional changes. Furthermore, MMP activity was sustained, thus shifting the remodeling balance of aged and induced‐senescent tissues towards degradation over production. Together, this demonstrates that cellular senescence plays a role in the altered mechano‐response of aged tendons and likely contributes to poor clinical outcomes in aging populations.

## Graphical abstract

We developed a novel in vitro model of induced cellular senescence in murine tendon explants and then utilized this model to compare ECM remodeling of young, aged, and induced‐senescent tendons to an altered mechanical stimulus. Both senescence and aging led to reduced protein synthesis with sustained MMP activity, thus shifting the remodeling balance towards degradation over production. This implicates senescence as a key player in altered mechanobiology of aged tendons and a likely contributor to poor clinical outcomes.

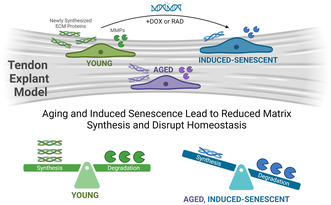

**Abbreviations**
ANOVA: Analysis of variance
BSA: Bovine serum albumin
Casp3: Caspase 3
D0: Day 0
DAPI: 4′,6‐diamidino‐2‐phenylindole
DMMB: Dimethyl methylene blue
DOX: Doxorubicin
ECM: Extracellular matrix
FDA: Fluorescein diacetate
FDL: Flexor digitorum longus
GAG: Glycosaminoglycan
IL: Interleukin
Lmb1: Lamin B1
MATL: Multi‐area time lapse
MIP: Maximum intensity projections
MMP: Matrix metalloproteinase
OHP: Hydroxyproline
PBS: Phosphate buffered saline
PI: Propidium iodide
RAD: Irradiation
ROI: Region of Interest
SASP: Senescence‐associated secretory phenotype
SA‐β‐Gal: Senescence‐associated beta‐galactosidase
sGAG: Sulfated glycosaminoglycan
SLRP: Small leucine‐rich proteoglycans
SP: Staurosporine
TBS: Tris buffered saline
TIMPs: Tissue inhibitors of MMPs

## 1 INTRODUCTION

Musculoskeletal injuries present a significant clinical burden for aging patients, accounting for 25%–30% total years lived with disability in elderly populations (Briggs et al., 2016). These injuries cause pain, decreased mobility, loss of independence, and reduced quality of life. Tendons are an important fibroelastic musculoskeletal tissue that connects muscle to bone, transmitting mechanical forces and storing energy to facilitate movement (Siadat et al., 2021). Tendon injuries are especially common in aging populations. Rotator cuff tears, one of the most common conditions, were found to affect more than 50% of people over 80 years of age (Teunis et al., 2014). In addition to the high prevalence of tendon injuries, aged patients often demonstrate poor healing outcomes characterized by high rates of reinjury (Ackerman et al., 2017). It has been documented that both acute tendon injuries and tendinopathies are associated with a chronic degenerative state. However, the progression and biological drivers of age‐related tendon degeneration are poorly understood.

Tendon function is highly dependent on its extracellular matrix (ECM) structure, composed of a fibrillar matrix, primarily type 1 collagen, as well as an interfascicle matrix containing proteoglycans, glycoproteins, and minor collagens (Siadat et al., 2021). While many studies have examined mechanical properties, tissue composition, and matrix organization of aged tendons, there is little consensus on specific age‐related changes, with results often depending on the tendon type and donor age (Siadat et al., 2021). Specifically, prior work investigating alterations in collagen content, fiber orientation, and crimp morphology in aged tendons has yielded conflicting results. However, there is supportive evidence for an increase of nonenzymatic crosslinking via advanced glycation end products in aged tendons (Couppé et al., 2009; Stammers et al., 2020). The number of collagen fascicles was also found to decrease with increasing age, suggesting an increased proportion of interfascicular matrix. However, interfascicular proteins, such as glycosaminoglycans (GAGs), were found to either decrease or show no differences with age. Consistent cellular changes, however, have been observed in aged tendons with decreased tissue cellularity and altered tendon cell morphology (Siadat et al., 2021). Furthermore, it is clear that aged tendons exhibit alterations in cellular activity and cell‐mediated processes such as decreased proliferation and metabolic activity (Connizzo et al., 2020; Siadat et al., 2021). This is supported by observations of impaired healing (Ackerman et al., 2017) and suggests a compromised ability of aged cells to regulate tissue homeostasis as well.

It is widely accepted that dysregulation of balanced ECM turnover plays a role in tendon degeneration. Under healthy conditions, primary tendon cells, tenocytes, are responsible for remodeling the ECM in response to changing mechanical loads, allowing the tissue to adapt and repair matrix microdamage (Siadat et al., 2021). This process is a tightly regulated yet delicate balance of matrix clearance and synthesis. Tenocytes produce matrix‐degrading enzymes, such as matrix metalloproteinases (MMPs) and other proteases, to clear damaged matrix (Sbardella et al., 2014). In parallel, cells synthesize new matrix proteins that are incorporated into the matrix and reorganized into functional tissue structure (Aggouras et al., 2024; Connizzo et al., 2020). The turnover rate of fibrillar collagen is relatively low, with only a small fraction of collagen present in tendon remodeling daily (Siadat et al., 2021). It has been hypothesized that pools of collagens in tendon have differential turnover rates. For example, small‐diameter type 1 fibrils, as well as non‐fibrillar collagens present in the interfascicle matrix, are more rapidly replaced than large‐diameter fibrils (Choi et al., 2020). These cell‐driven adaptations also allow the tissue to meet changing mechanical demands. Both exercise and disuse have been reported to initiate ECM remodeling, with sustained exercise reportedly increasing tendon mechanical strength and matrix synthesis (Rooney et al., 2017) and disuse resulting in a decline in mechanical properties (Couppé et al., 2012). However, biological factors that alter cellular processes, such as aging or disease, can lead to dysregulation of ECM remodeling and put tissues at increased risk for injury.

While there are several well‐established cellular changes associated with aging (López‐Otín et al., 2023), one of the most promising candidates implicated in disrupting ECM remodeling is cellular senescence. Cellular senescence is a permanent state of cell arrest in response to external damage stimuli or stressors (López‐Otín et al., 2023). Senescent cells are documented to be resistant to apoptosis and accumulate in many aged tissues, including tendon (Hawthorne et al., 2023; Kohler et al., 2013), likely due to impaired immune clearance with aging. Importantly, the senescence‐associated secretory phenotype (SASP), which has been reported to include pro‐inflammatory cytokines and MMPs, has the potential to degrade tissue matrix and induce neighboring cells to a senescent phenotype via secondary senescence (Coppé et al., 2010). While senescence is essential in development, protection against cancers, and wound repair, senescent cells are documented to have a negative effect during aging, leading to a decline of regenerative capacity, increased inflammation, and loss of function. More recently, senescent cells have been implicated in degenerative musculoskeletal diseases, such as osteoarthritis (Coryell et al., 2021).

Much of what we know about senescent cells and their biomarkers comes from isolated cell culture, including the first descriptions of senescence by Hayflick almost 60 years ago (Hayflick, 1965). In vitro induction of premature cellular senescence has been informative in identifying and characterizing the phenotype of senescent cells, as well as screening potential senotherapies that selectively target senescent cell populations (Chaib et al., 2022). Senescence can be induced in vitro with various stress or damage stimuli including extensive replication, oxidative stress, oncogene activation, mitochondrial dysfunction, and DNA damage (González‐Gualda et al., 2021). Commonly used DNA‐damaging agents to induce senescence in vitro include radiation and chemotherapy drugs, such as doxorubicin (Copp et al., 2021; Kirsch et al., 2022). Senescence has been induced previously in rat patellar tendon stem cells (Nie et al., 2021), murine fibroblasts (Saito et al., 2020), and primary human tenocytes (Poulsen et al., 2014). However, as 2D cell culture alone does not replicate native cell–cell and cell–matrix connections necessary for studying ECM remodeling, the link between cellular senescence and dysregulation of ECM maintenance has not yet been elucidated.

Our lab utilizes a unique model of live tendon explants to directly assess ECM remodeling in real time while maintaining intact cell‐ECM interactions. This novel technique enables the study of local cell biology in a more physiologically relevant context without interference from age‐related systemic changes, such as immune responses and metabolic disease. Previous work by our group investigated age‐related changes of murine flexor digitorum longus (FDL) tendon explants in response to an altered mechanical stimulus (Connizzo et al., 2020). Despite no age‐related changes at baseline, we found that aged tendons exhibited reduced metabolism, proliferation, and matrix synthesis in response to mechanical unloading, indicating altered mechanoresponses with aging. Accompanying this, we found an increase in senescence‐associated gene expression (p16, p19, and p53) in aged tendons. While additional studies by our lab and others have suggested a role for senescent cells in age‐related ECM pathology, the ability of senescent cells to perform functional matrix remodeling is still largely unknown.

Therefore, the overarching goal of this study was to directly investigate the role of senescent cells in the dysregulation of tendon ECM homeostasis. Our first aim was to develop a model of induced cellular senescence in both primary murine tenocytes and flexor tendon explants to characterize the senescent phenotype of tendon cells and study the unique signature of senescent tenocytes within their native matrix environment. Utilizing our newly developed in vitro senescent explant model, our secondary objective was to compare the matrix remodeling response of young, naturally aged, and induced‐senescent tendons to an altered mechanical stimulus. We hypothesized that both aged and senescent tendons would exhibit altered ECM remodeling following a mechanical unloading injury, with a shift to processes that promote ECM degradation over synthesis.

## 2 METHODS

### 2.1 Sample Preparation

**FIGURE 1:**
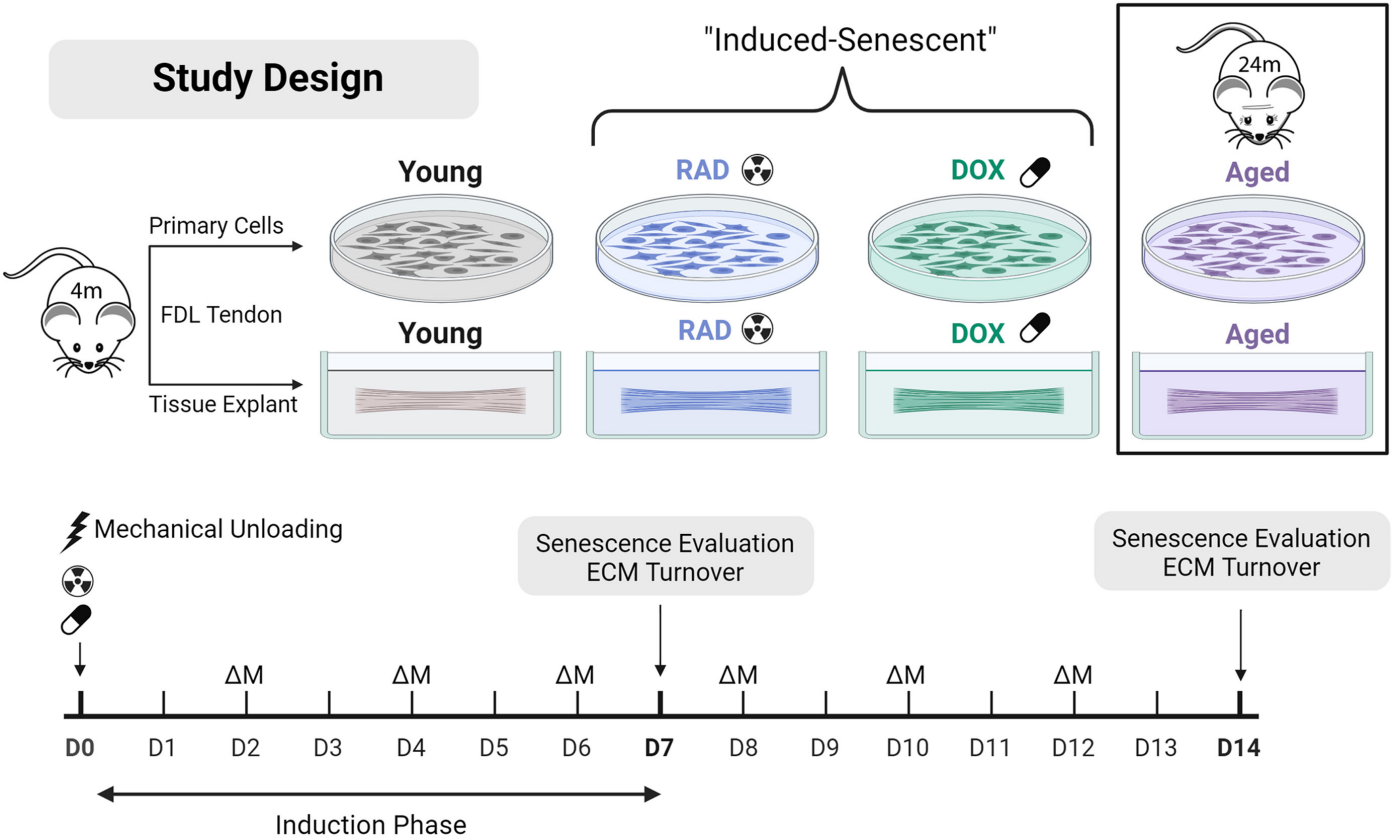
Experimental design. This study aimed to develop a model of induced cellular senescence in tendon explants (Aim 1) and investigate the impact of senescent cell populations on tendon ECM remodeling (Aim 2). Primary cells and tendon explants were harvested from flexor digitorum longus (FDL) tendons of young (4‐month‐old), mice and senescence was induced in young cells and tendon explants using doxorubicin (DOX) and irradiation (RAD) treatment. Tendons and cells were also harvested from aged (24‐month‐old) mice for comparison. Study timeline is shown at the bottom of the figure. Extraction from physiological loading at harvest results in a mechanical unloading injury to tendon explants. Senescence induction and ECM turnover were evaluated after 7 and 14 days of culture. Media changes (ΔM) were performed every 2 days. Created with BioRender.com.

Primary tenocytes and tendon explants were obtained from FDL tendons harvested from 4 month old (young) and 20–24‐month old (aged) male C57BL/6J mice. Aged mice were obtained from the NIH/NIA Aging Rodent Colony, and young mice were obtained from Jackson laboratories. Primary FDL murine tenocytes were isolated as described previously (Paredes et al., 2020). Briefly, tendons were dissected, quickly dipped in 70% ethanol to kill surface cells, minced, and digested with 2 mg/mL of collagenase type I (Gibco) and 1 mg/mL of collagenase type IV (Worthington Biochemical) in low‐glucose DMEM for 2 h at 37°C on a rocking shaker. Ten FDLs were pooled for each biological replicate. Cell suspensions were filtered with a 70‐μm cell strainer, plated at P0 in a T‐25 culture flask at 1200 cells/cm^2^, and expanded in culture until P1 when senescence was induced. FDL tendon explants were harvested as described previously (Connizzo et al., 2020). After tissue extraction, explants were washed in 1× PBS supplemented with antibiotics and placed in culture medium. For both cells and explants, culture medium consisted of low glucose Dulbecco's Modified Eagle Media (DMEM) (1 g/L (Fisher Scientific)) with 10% fetal bovine serum (Cytiva), 100 units/mL penicillin G and 100 μg/mL streptomycin (Fisher Scientific). Culture medium was replaced every 2 days of culture. Tendon explants were cultured under stress deprivation (Connizzo et al., 2020), where tendons were free floating in media without any mechanical loading for up to 14 days (Figure 1).

### 2.2 Senescence induction

In vitro cellular senescence was induced in tendon explants and primary tenocytes with two methods: irradiation (RAD) and doxorubicin (DOX) (Figure 1). For RAD treatment, a 10Gy dose of irradiation was delivered at Day 0 of culture using an X‐ray irradiator (Precision X‐Ray) (Copp et al., 2021; Noren Hooten & Evans, 2017). For DOX treatment, explants and cells were cultured in 200 nM doxorubicin hydrochloride (Sigma) in culture medium for the first 72 h, after which they were switched to standard medium for the remaining culture period (Kirsch et al., 2022). Senescence was evaluated after 7 days, and additional assays were performed at day 14 to confirm a sustained phenotype. It is well established that there is no universal biomarker for cellular senescence. Therefore, we evaluated senescence with a panel of biomarkers including DNA damage (γH2AX, Section 2.6), cell cycle arrest (thymidine incorporation, Section 2.4, and gene expression of cell cycle regulators, Section 2.5), secretory phenotype (protein secretion in media, Section 2.8), apoptosis resistance (Section 2.7), metabolic changes (Section 2.3), increased lysosomal content (SA‐β‐Gal expression, Section 2.6), and nuclear changes (loss of nuclear protein lamin B1, Section 2.5).

### 2.3 Explant viability and metabolic activity

Explant viability was assessed using live/dead imaging (*n* = 4/group). Whole explants were incubated for 5 min in 1× PBS with fluorescein diacetate (FDA; 4 mg/mL, Sigma) and propidium iodide (PI; 1 mg/mL, Sigma), to label viable and nonviable cells, respectively. Samples were mounted between two pieces of #1.5 coverslip with a small layer of 1× PBS and imaged via confocal microscopy (10×, Olympus FV3000). Multi‐area time lapse (MATL) software was used to scan across the desired region of interest to form a full tendon viability map. Z‐stacks of approximately 80–100 μm thickness were generated, and maximum intensity projections (MIPs) were formed. The total number of live and dead cells was quantified with a custom MATLAB script that thresholds channels individually and analyzes the total number of cells in a 7 mm region of interest (ROI). Viability is expressed as a percentage of number of live cells divided by total number of cells in the specified ROI. Cell density is calculated as the total number of cells divided by the ROI area.

Explant and cell metabolism were assessed throughout culture with a resazurin reduction assay (Connizzo et al., 2020). Samples (*n* = 5/group/time) were incubated with media containing 10% v/v resazurin. After 3 h, fluorescence intensity in spent medium was measured at 554/584 nm. Data is expressed normalized to control medium with resazurin, such that a value of ‘1’ represents no detectable metabolic activity.

### 2.4 Cell proliferation, matrix biosynthesis, and matrix composition

Synthesis rates of sulfated glycosaminoglycans (sGAG), total protein (indicative of collagen synthesis), and DNA (cell proliferation) were measured via incorporation of radiolabeled sulfate, proline, and thymidine, respectively. Radiolabels ^35^S‐sulfate (2 μCi/mL), ^3^H‐proline (1 μCi/mL), and ^3^H‐thymidine (1 μCi/mL) (Perkin‐Elmer) were added directly to culture medium for 24‐h (proline and thymidine radiolabeling performed in separate experiments). Following radiolabel incorporation, tendon wet weights were measured after soaking the tendon in 1× PBS for 1‐min. Tendons were then lyophilized for 3‐h, and tendon dry weights were recorded. Water content of the tendon was calculated as the difference in wet weight and dry weight divided by the dry weight and multiplied by 100. Tendons (*n* = 5/group/time) were then digested with proteinase‐K. (5 mg/mL) (Sigma) for 18 h and stored at −20C until further assays could be performed. Cell suspensions (*n* = 5/group) were lysed with sodium hydroxide and stored at −20C. Radiolabel incorporation was measured with a liquid scintillation counter (Perkin–Elmer) and values represent the rate of incorporation over the 24‐h time period. Incorporation rates for each explant sample are normalized to the tissue dry weight to account for variability in sample size. Incorporation rates for cells are normalized to the total number of cells.

Additional biochemical assays were then performed with the digest to quantify explant composition (Connizzo et al., 2020). sGAG content was measured using the dimethyl methylene blue (DMMB) assay. Double‐stranded DNA content was measured using the PicoGreen dye binding assay. A 100 μL portion of each digest was then hydrolyzed using 12 M HCl, dried, resuspended, and assayed to measure total collagen content using the hydroxyproline (OHP) assay.

### 2.5 Quantitative gene expression

Explants from each group were collected at day 0 (baseline), day 7, and day 14 (*n* = 5/group/time). Explants were immediately flash frozen with liquid nitrogen and stored at −80°C until RNA extraction, performed as described previously (Grinstein et al., 2018). Total RNA was then reverse‐transcribed to cDNA, and real‐time polymerase chain reaction (PCR) was performed using the Applied Biosystems StepOne Plus RT‐PCR with SYBR Green Master Mix (Applied Biosystems). Murine gene names and primer sequences are listed in the (Table S1). Briefly, we measured markers of cell cycle (p16^ink4a^, p53, and p21), nuclear membrane (lamin B1), apoptosis (caspase‐3), inflammation (IL‐6, CXCL1, IL‐1β), matrix metalloproteinases (MMP‐1, MMP‐3, MMP‐13), and ECM proteins (collagen 1, decorin, fibromodulin). Expression for each gene was calculated from the threshold cycle (C _t_) value, and fold changes were calculated by normalizations to the housekeeping gene, beta‐actin, and to day 0 values using the double delta CT method (Livak & Schmittgen, 2001). All data are presented in log space.

### 2.6 Histological assessment

Samples were analyzed for SA‐β‐Gal, γH2AX, and p21 protein using standard immunohistochemical techniques. Fresh tendons (*n* = 4–5/group) were removed from culture, embedded in OCT media, and flash frozen. Frozen samples were transferred to −20°C until sectioning. SA‐β‐Gal samples were stored for a maximum of 72‐h before sectioning and staining to prevent loss of activity. Tendons were cryosectioned with a 10 μm thickness at −20°C. Cryosections were allowed to briefly air dry before being rehydrated with a graded ethanol series prior to staining. Cells were plated on 12 mm #1 glass coverslips pre‐coated with poly‐d‐lysine (Neuvitro Corporation) at a seeding density of 75,000 cells per 24‐well, allowed to adhere overnight, and fixed for 10 min at room temperature with 2% paraformaldehyde and washed with 1% BSA to remove fixation solution.

SA‐β‐Gal was stained using CellEvent™ Senescence Green Detection Kit (Invitrogen), following manufacturer guidelines. Immunostaining for γH2AX (phospho‐H2AX (Ser139) Alexa Fluor 647 conjugate antibody, Cell Signaling, 1:200 dilution) and p21 (rabbit monoclonal to p21(ab188224), 1:500 dilution; goat anti‐rabbit IgG H&L (Alexa Fluor® 647) (ab150079), 1:200 dilution) was performed using standard immunostaining protocols. Cell coverslips were permeabilized for 10‐min in 0.2% Triton X‐100 prior to a 1‐h block in 5% normal goat serum. Tissue sections were exposed to combined blocking and permeabilization solution (0.2% Triton X‐100 and 5% normal goat serum in PBS) for 1‐h prior and throughout the overnight antibody incubation steps. TBS was used in place of PBS for detection of phosphoproteins (γH2AX). Stained tissue sections and cell coverslips were mounted with DAPI antifade mounting media (ProLong™ Gold Antifade Mountant with DAPI, Life Technologies) and imaged on an inverted microscope (20×, Olympus IX83) with appropriate filters (DAPI/FITC/Cy5). Small Z‐stacks were collected for tissue sections and 3D deconvolution was performed to improve resolution and contrast. Background‐subtracted MIPs were analyzed using a custom MATLAB script that thresholds channels individually using a global threshold value and counts the number of cells in the FITC and Cy5 channels that co‐localize with DAPI signal. In cells, individual γH2AX foci could be resolved, and the number of foci per nuclei was quantified by counting puncta within each thresholded DAPI area. Since SA‐β‐Gal and γH2AX were co‐stained, dual staining was quantified by co‐localizing DAPI with both FITC and Cy5 signals.

### 2.7 Apoptosis resistance

Apoptosis was induced with 1 μM staurosporine (SP) treatment for 24‐h. Repeated resazurin reduction assays were performed before and after treatment such that cell death due to SP could be directly monitored for each sample (*n* = 5/group). Percent loss in metabolism between repeated assays was calculated as a measure of cell death, where reduced cell death (reduced loss in metabolism) represents a resistance to apoptosis.

### 2.8 Secretory profile analysis

Activity of MMPs (1, 2, 3, 7, 8, 9, 10, 13, and 14) was determined via analysis of spent culture medium (*n* = 5/group/time) using a commercially available FRET‐based generic MMP activity kit (SensoLyte 520 Generic MMP Activity Kit Fluorimetric; Anaspec). MMP activity is represented as the concentration of MMP‐cleaved product (5‐FAM‐Pro‐Leu‐OH), the final product of the MMP enzymatic reaction. Secretion of SASP‐associated proteins was assessed in spent media samples (*n* = 5/group/time) using a custom multiplex immunoassay (UPLEX Biomarker Group 1 (mouse); Meso Scale Discovery) for 10 analytes: GM‐CSF, IFN‐γ, IL‐1β, IL‐6, IL‐10, IL‐13, KC/GRO (CXCL1), MCP‐1, MIP‐1α, and TNF‐α.

### 2.9 Tensile loading bioreactor studies

To remove confounding effects of stress deprivation, senescence induction with doxorubicin (Section 2.2) was performed under 3% cyclic tensile strain. As published previously, tendon explants were gripped and loaded into an incubator‐housed tensile loading bioreactor (Figure S5) (Aggouras et al., 2024). Explants were preloaded to 20 g and loaded using a displacement‐controlled waveform at 3% strain at 1 Hz for 1 h followed by a 5‐h hold. This protocol was repeated four times a day for the duration of the 7‐day culture period. Senescence evaluation was performed as described above on day 7.

### 2.10 Statistical evaluation

All data are presented as mean ± 95% confidence interval. Data points more than two standard deviations from the mean were removed as outliers. Statistical evaluation was performed using one‐way analysis of variance (ANOVA) at each timepoint. Tukey post hoc *t*‐tests were then used to identify differences within each time point where appropriate. While multiple comparisons were performed between all experimental groups, only significant comparisons to the young control group are shown on plots for simplicity. All comparisons and statistical significance values are presented in the Appendix S1. For all comparisons, significance is noted with **** for *p* < 0.0001, *** for *p* < 0.001, ** for *p* < 0.01, and * for *p* < 0.05. Trends are shown with dotted lines (*p* < 0.1). Unpaired *t*‐tests were performed to assess differences from day 0 baseline data, where significant differences (*p* < 0.05) from day 0 are noted with the hashtag (#) symbol.

## 3 RESULTS AND DISCUSSION

The objectives of this study were twofold: (1) to establish a model of induced cellular senescence in tendon explants, and (2) to use this model to investigate how aging and cellular senescence independently affect the capacity for cell‐driven matrix remodeling (Figure 1).

### 3.1 DOX and RAD induce senescence in primary murine tenocytes

**FIGURE 2:**
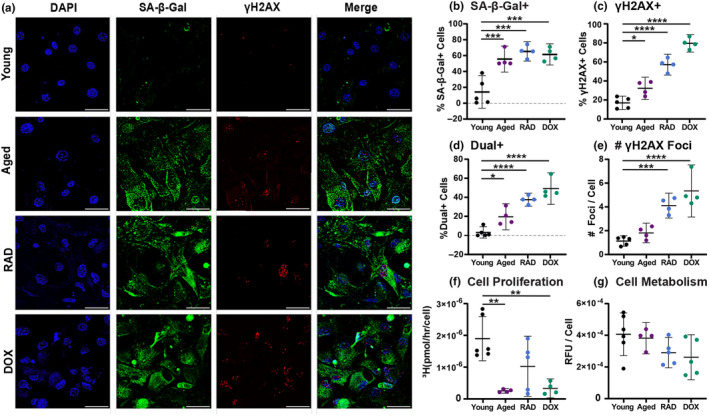
Senescence Induction in Murine Tenocytes. (a) Representative images of young, aged, RAD, and DOX cells stained with DAPI (blue), SA‐β‐Gal (green), and γH2AX (red). Associated image quantification for (b) SA‐β‐Gal^+^ cells, (c) γH2AX^+^ cells, (d) dual label^+^ cells, and (e) number of γH2AX foci per cell. (f) Total cell proliferation and (g) metabolic activity. Scale bars are 50 μm. Significant statistical comparisons to young control cells are shown with **** for *p* < 0.0001, *** for *p* < 0.001, ** for *p* < 0.01, and * for *p* < 0.05. Trends (*p* < 0.1) are shown with dashed lines.

We started by inducing cellular senescence in 2D cell culture, as this is more commonly done in the literature (Copp et al., 2021; Kirsch et al., 2022; Nie et al., 2021; Poulsen et al., 2014). Senescence is typically evaluated with a panel of general hallmarks and biomarkers including DNA damage (ex. γH2AX), cell cycle arrest, a secretory phenotype, apoptosis resistance, metabolic changes, morphological changes, SA‐β‐Gal expression, and nuclear changes (González‐Gualda et al., 2021). It is well established in the field that there is no universal biomarker for cellular senescence and the induced‐senescent phenotype appears heterogeneous and dynamic across cell types and induction stimuli (Hernandez‐Segura et al., 2018). Due to this, a concurrent validation of multiple hallmarks is recommended to confirm a senescent phenotype in vitro. Cellular senescence was induced in primary murine tenocytes using 10Gy radiation and 200 nM doxorubicin treatment (Figure 2). Image quantification revealed increased SA‐β‐Gal^+^ and γH2AX^+^ cell populations in both the aged and induction groups (RAD and DOX) compared to the young cells (Figure 2b–d). Furthermore, both induction groups had a larger number of γH2AX foci per cell, indicating increased accumulation of irreparable DNA damage (Figure 2e). Significant decreases in cell proliferation were found in aged and DOX cells when compared to control cells, and although not significant and highly variable, RAD cells also exhibited a reduction in proliferative capacity (Figure 2f).

Our approach resulted in an induction efficiency of 37% for RAD and 49% for DOX‐treated cells, based on the percentage of dual‐positive cells. Reported induction efficiencies in the literature vary, with over 75% of rat patellar tendon cells after treatment with bleomycin (Nie et al., 2021) and around 30% of human tenocytes following dexamethasone treatment (Poulsen et al., 2014). Importantly, we found low baseline levels of senescence (3%) in young tenocytes and elevated levels (20%) in aged tenocytes. In addition, the number of aged cells staining positive for SA‐β‐Gal is similar to what has been documented previously in the literature in tendon stem cells (M. Chen et al., 2020). Together, this demonstrates that induced‐senescent tenocytes exhibit common biomarkers of cellular senescence (SA‐β‐Gal, γH2AX, and cell cycle arrest) that are consistent with previous studies (Cai et al., 2022; Nie et al., 2021; Poulsen et al., 2014).

### 3.2 DOX and RAD induce senescence in murine tendon explants

**FIGURE 3:**
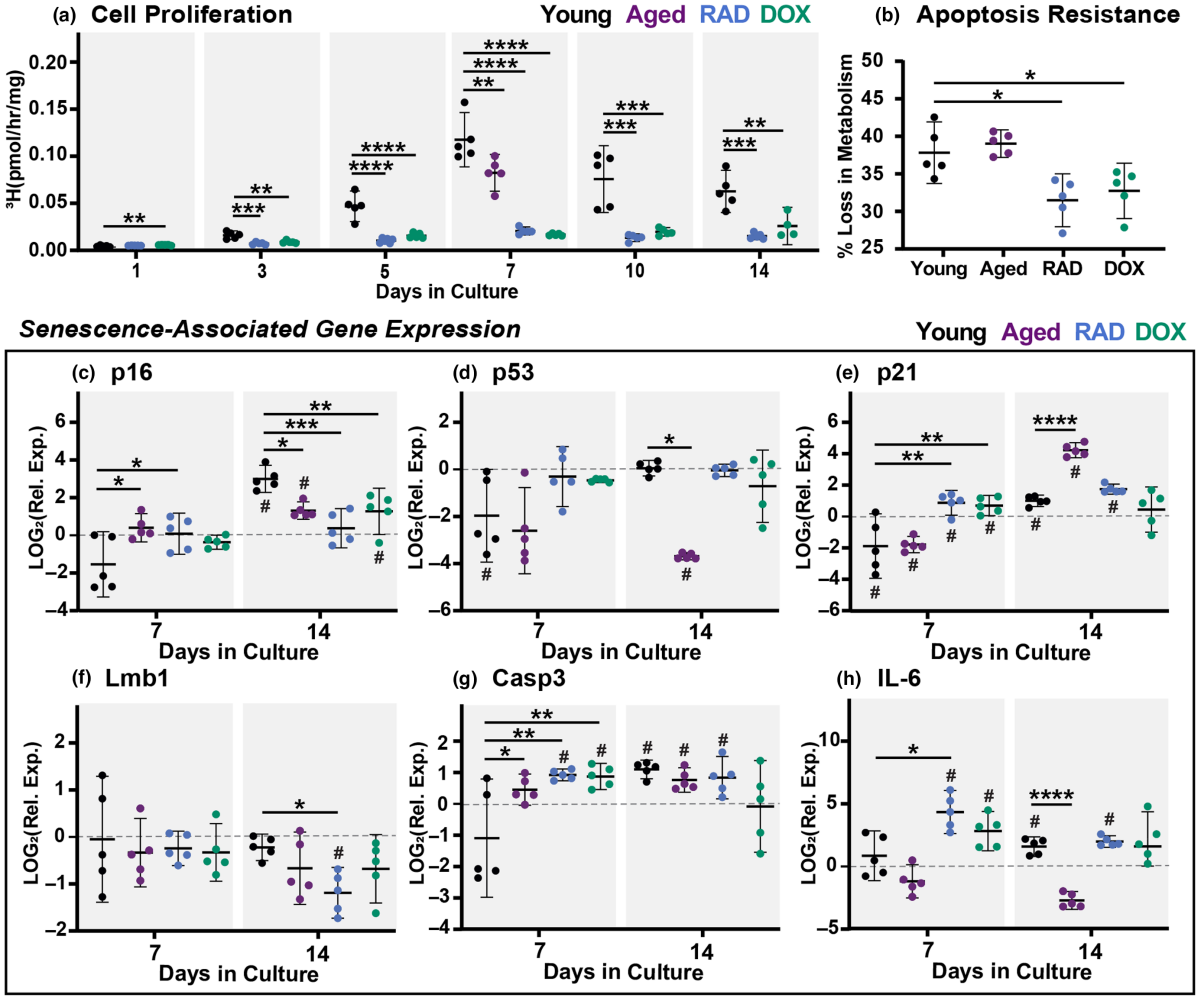
Senescence Induction in Tendon Explants. (a) Cell proliferation at days 1, 3, 5, 7, 10, and 14. Aged data available for day 7 only. (b) Apoptosis resistance at day 7 shown as percent loss in metabolism following 24‐hr treatment with staurosporine. Senescence‐associated gene expression at day 7 and day 14 for (c) p16, (d) p53, (e) p21, (f) Lmb1, (g) Casp3, and (h) IL‐6. Gene expression data presented as relative expression to D0. Significant statistical comparisons to young explants are shown with **** for *p* < 0.0001, *** for *p* < 0.001, ** for *p* < 0.01, and * for *p* < 0.05 (solid lines). Trends (*p* < 0.1) are shown with dashed lines. # indicates significant differences from day 0 values (*p* < 0.05).

We next aimed to induce cellular senescence in tendon cells within their native ECM. Using the same induction methods (RAD and DOX) with a slightly larger panel of assessment biomarkers, we have documented successful induction of cellular senescence in live murine tendon explants. First, we confirmed all groups were viable following treatment (Figure S1). We then assessed a time course of cell proliferation as a metric of cell cycle arrest. For a young explant under stress deprivation, proliferative capacity increased throughout culture until day 7 (Figure 3a). Compared to the young tissues, both DOX‐ and RAD‐treated explants exhibited significantly reduced proliferation (Figure 3a), starting at day 3 and continuing through day 14. Proliferation of aged tissues was only assessed at day 7 but was also found to be significantly reduced compared to young tissues. At day 7, RAD and DOX tendons also exhibited significant reductions in metabolic loss following staurosporine treatment, indicating resistance to apoptotic cell death (Figure 3b). Conversely, aged tendons did not show significant apoptosis resistance. Together, this demonstrates arrested growth and apoptosis resistance in induced‐senescent groups, and to an extent in aged groups, consistent with the presence of senescent cell populations.

Senescence‐associated gene expression was then assessed at days 0, 7, and 14 (Figure 3c–h). In young tissues, expression of p16, p53, p21, casp3, and IL‐6 appears to increase over time in culture. We suspect this represents part of the injury response to stress deprivation, resulting in changes in cell health, proliferation, and apoptosis (Egerbacher et al., 2008). Looking more closely at group differences within timepoints, p16 was found to be increased in aged and RAD tendons compared to young tendons at day 7 (Figure 3c). At day 14, young explants have increased p16 expression compared to all other groups. Expression of p53 was downregulated from baseline in young explants at day 7, but returned to baseline by day 14 at which point aged tissues demonstrated reduced p53 expression (Figure 3d). Interestingly, p21 was downregulated at day 7 in young and aged explants but upregulated in the RAD and DOX groups, resulting in significant differences between groups (Figure 3e). By day 14, aged tendons had highly upregulated p21 expression compared to young tendons. RAD tendons exhibited a downregulation in lmb1 compared to young tendons at day 14 (Figure 3f). Expression of casp3 was increased for aged, RAD, and DOX explants compared to the young group at day 7 (Figure 3g), but increases in the young group between days 7 and 14 resulted in no significant differences between groups later on. IL‐6 was found to be upregulated for RAD and DOX explants at day 7 (Figure 3h). However, again, these differences were lost by day 14 when young explants exhibited upregulation of IL‐6 relative to baseline. Aged tendons exhibit significantly reduced expression of IL‐6 compared to young tendons at day 14. It's critical to point out here that DOX and RAD groups exhibit reduced differences over time in culture in all measured parameters, suggesting a sustained phenotype throughout culture. Aged tendons also do not appear to change over time, with the exception of p21 expression, which increases. This could suggest a population of senescent cells present in aged tissues at the onset of the culture period and potentially a growth of this population through mechanical injury.

**FIGURE 4:**
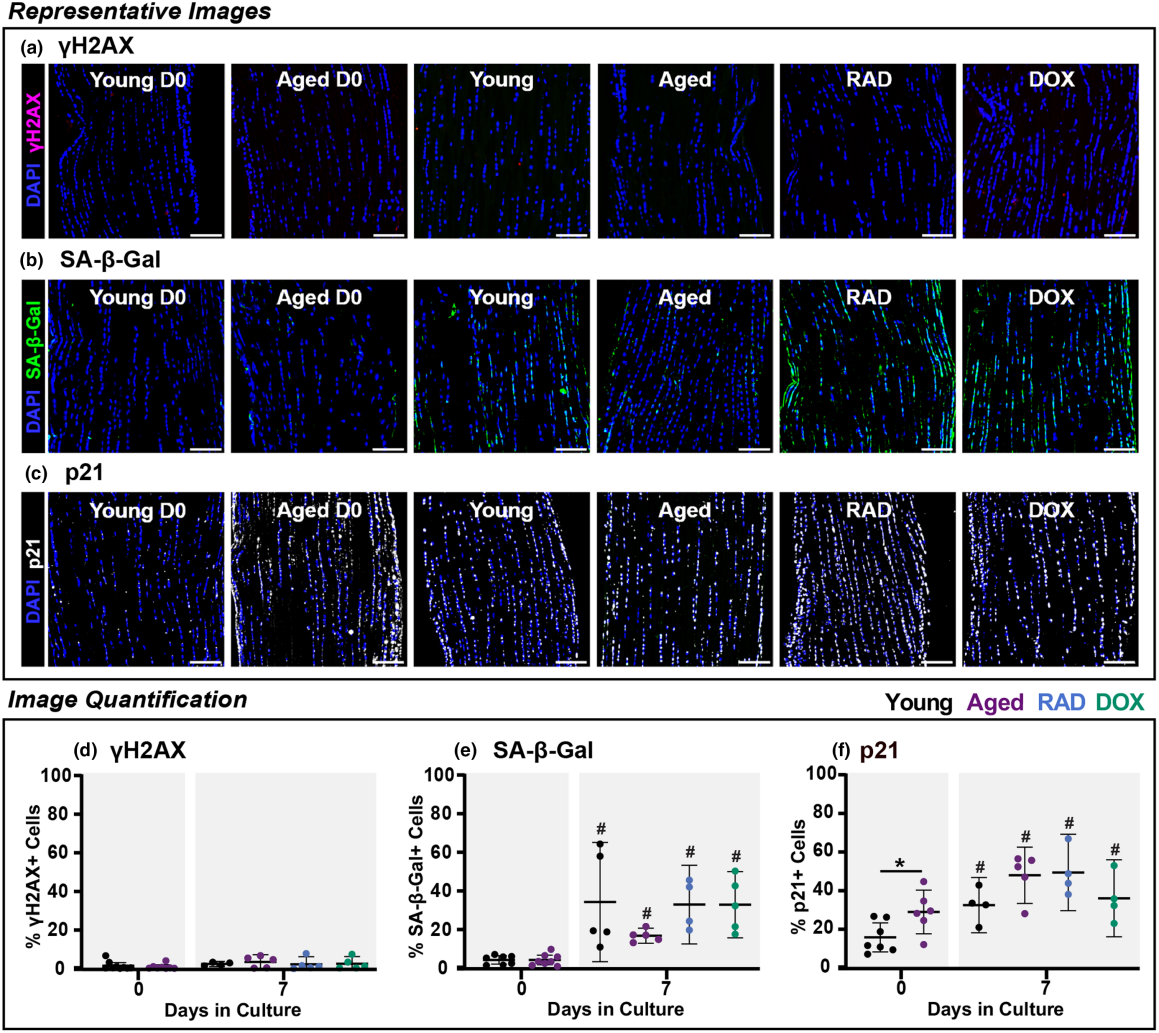
Senescence Induction in Tendon Explants. Representative images of tendon explants stained with DAPI (blue) and (a) γH2AX (magenta), (b) SA‐β‐Gal (green), and (c) p21 (white) at day 7. Scale bars are 100 μm. While little γH2AX staining was found in all experimental groups, positive control (2 h following irradiation) demonstrated robust staining and is included with supplemental materials. Associated image quantification for (d) γH2AX, (e) SA‐β‐Gal, and (f) p21. Significant statistical comparisons to young explants are shown with * for p < 0.05.

We then sought to confirm the expression of senescence‐associated proteins in our study groups via histological assessment for γH2AX, SA‐β‐Gal, and p21 at days 0 and 7. Surprisingly, very few γH2AX‐positive cells were found (<10%+) for all groups at both day 0 and day 7 (Figure 4d). However, large populations of H2AX‐positive cells (>75%+) were found in explants exposed to 10Gy irradiation and incubated for 2‐h before takedown (Figure S2), an established experimental positive control (Copp et al., 2021). Quantification revealed significant increases SAβ‐Gal between day 0 and day 7 for all groups as well (Figure 4e). However, no significant differences were found at day 7 between control, aged, or induced‐senescent groups.

Quantification of p21 staining also showed increased p21 protein expression between days 0 and 7 for all groups (Figure 4f). Aged and RAD tendons appear to have increased number of p21+ cells compared to young tissues at day 7 but significance could not be detected with this sample size. Combined with our gene expression data, this could suggest p21 as a key marker of cellular senescence at this timepoint in our model system.

Together, we document substantial reductions in cellular proliferation, resistance to apoptotic cell death, elevated expression of cell cycle regulators, reduced expression of nuclear proteins, and increased expression of SASP factors in induced‐senescent tendon explants. Comparing native tendons from young and aged mice, we also document increased expression of senescence‐associated genes in aged tissues (p16, p53, p21, IL‐6, MMP‐1, and MMP‐3) (Figure S3) as well as an increased number of p21‐positive cells in aged tissues at baseline (Figure 4f). This suggests that aged tendons contain senescent cells and that young tendon cells can be induced to senescence with various stress stimuli. By comparing the senescence induction efficiencies and phenotypes between primary cells and tendon explants, we can assess the role of the extracellular matrix in the induction of cellular senescence. While we did not assess the exact same markers in cells and tissue explants, we do see SA‐β‐Gal expression and reduced proliferation in both model systems. Our quantification showed 60% SA‐β‐Gal^+^ cells and high expression of γH2AX (>60%) in induced primary tenocytes and less than 40% of SA‐β‐Gal^+^ cells and little γH2AX (<10%) in induced tendon explants. This could suggest a potential protective mechanism of the ECM towards senescence induction that has not been explored due to the lack of tissue induction studies. It's also possible that γH2AX would be present later in culture, and future studies will assess long‐term stability of the phenotype. Furthermore, we detected significant DNA damage 2‐h after irradiation exposure as in previous studies, but this damage was effectively repaired by 7 days in culture, potentially signifying an aid of the ECM in maintaining healthy cell processes.

### 3.3 Senescence impairs the capacity for ECM synthesis

**FIGURE 5:**
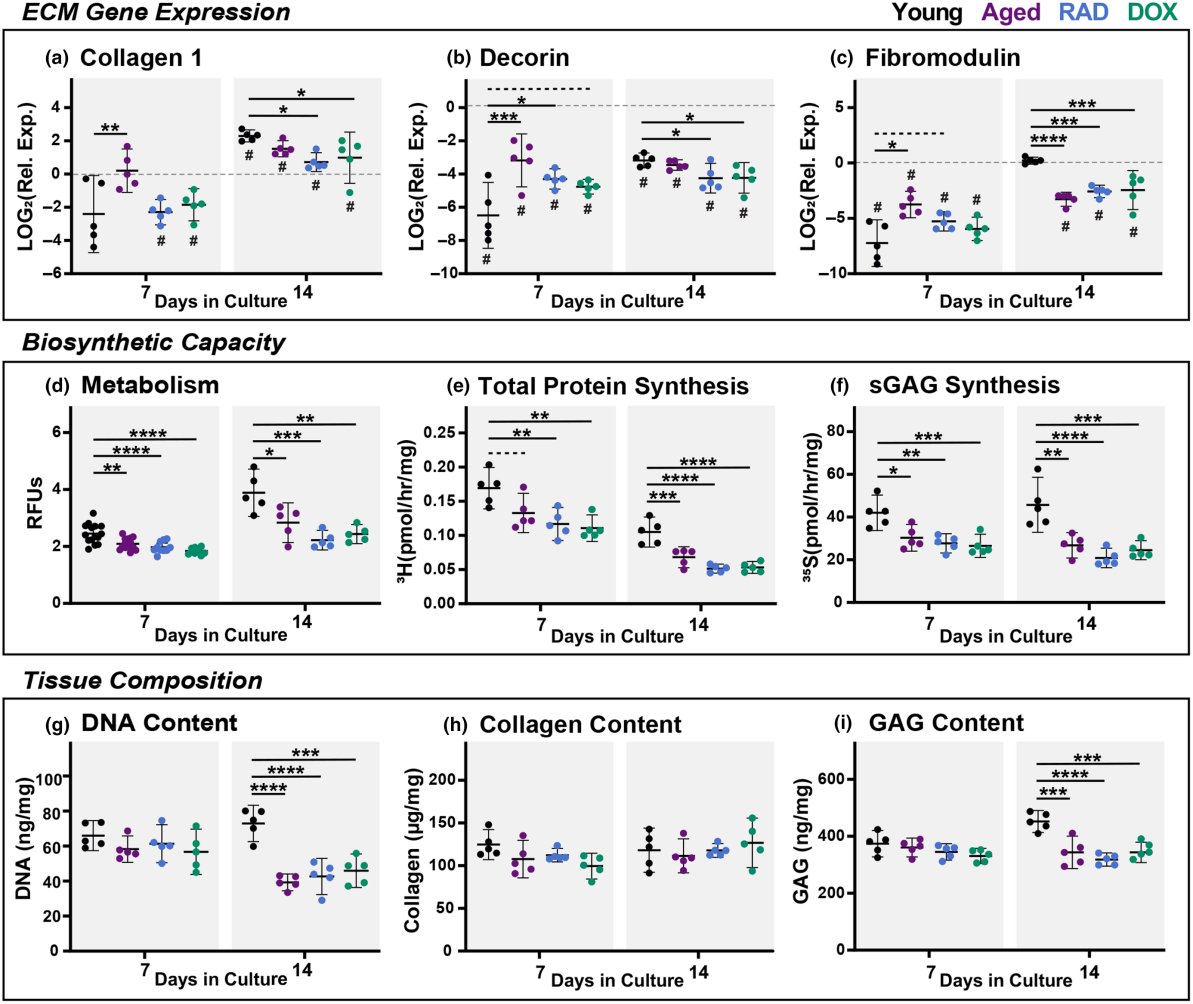
Matrix Synthesis and Composition. Relative gene expression of (a) collagen 1, (b) decorin, and (c) fibromodulin. Biosynthetic capacity assessed via (d) explant metabolic activity and ECM synthesis rates for (e) total protein and (f) sGAGs. Tissue composition of (g) DNA, (h) collagen, and (i) GAG. All data at day 7 and day 14. Gene expression data presented as relative expression to D0. Significant statistical comparisons to young explants are shown with **** for *p* < 0.0001, *** for *p* < 0.001, ** for *p* < 0.01, and * for *p* < 0.05 (solid lines). Trends (*p* < 0.1) are shown with dashed lines. # indicates significant differences from day 0 values (*p* < 0.05).

After characterizing our induced‐senescent explant model, we then began to assess how cellular senescence and natural aging independently affect the capacity for ECM remodeling following a mechanical unloading injury. At baseline, aged tendons exhibited reduced collagen 1 expression compared to young tendons (Figure S3). After 7 days in culture, collagen 1 expression was significantly downregulated from baseline for RAD and DOX explants, but it was no different from day 0 for young and aged tendons (Figure 5a). By day 14, collagen 1 expression was upregulated from baseline in all groups, and induced‐senescent groups exhibited significant decreases in expression compared to the young group. Together, this data suggests a sustained reduction of type 1 collagen expression in induced‐senescent tissues, signifying a reduced capacity to produce collagen necessary for homeostasis and repair. This is supported in the literature, where increased expression of collagen 1 was observed in aged tendons after treatment with a senolytic therapy (Hawthorne et al., 2023).

We also report differential gene expression profiles of small leucine‐rich proteoglycans (SLRPs) decorin and fibromodulin between young, aged, and induced‐senescent tissues. At baseline, aged tendons expressed reduced fibromodulin and decorin compared to young tendons (Figure S3). In young tendons, decorin and fibromodulin are initially downregulated, but expression increases over time in culture leading to dramatic changes between 7 and 14 days for both genes (Figure 5b,c). It's thought that this change is an adaptation to stress deprivation over the 2‐week culture period (Aggouras et al., 2024). In contrast, aged, RAD, and DOX groups exhibit consistently reduced expression throughout the culture period compared to baseline. This difference leads to relatively increased expression early in culture (day 7) and relatively decreased expression later in culture (day 14) compared to young tissues. Overall, this data suggests that the adaptive response to stress deprivation is lost in aged and induced‐senescent tissues. As previous work has established SLRPs as fundamental mediators of tendon healing and repair (Dunkman et al., 2014), as well as regulation of collagen structure, senescence‐associated proteoglycan loss could signify inferior tissue remodeling outcomes.

We then sought to identify if these changes in gene expression resulted in deficiencies in protein synthesis. At both day 7 and day 14, explant metabolism was reduced in aged, RAD, and DOX groups compared to the young explants (Figure 5d). The interactions of metabolic processes with both senescence and ECM remodeling are heavily studied areas of research (Sullivan et al., 2018; Wiley & Campisi, 2016). These complex processes are largely outside of the scope of these current findings, but we recognize altered metabolic activity is commonly observed with cellular senescence and could contribute to ECM remodeling outcomes. Aged and induced‐senescent explants also had significantly lower total protein synthesis compared to young tendons at both day 7 and day 14 (Figure 5e), as measured by proline incorporation. While this technique is not an exact measure of collagen synthesis, proline and hydroxyproline are the major amino acids present in type 1 collagen and are vital for procollagen synthesis, and thus it is a widely accepted corollary (Siadat et al., 2021). Reduced collagen synthesis could signify inadequate repair of microdamage and an inability to adapt to changing mechanical demands. Importantly, sGAG synthesis was also decreased for aged and induced‐senescent tendons at days 7 and 14 compared to young tendons (Figure 5f), further confirming the importance of disruption to non‐fibrillar turnover in aging tendons. Published work in 2D cell culture supports our findings, with both collagen and proteoglycan synthesis reportedly decreasing in induced‐senescent fibroblasts (Shelton et al., 1999; Takeda et al., 1992). In addition, induced‐senescent fibroblasts in an in vitro 3D tissue formation model exhibited reduced collagen fiber deposition and delays in tissue formation (Brauer et al., 2022).

Finally, we sought to identify how deficiencies in matrix synthesis in aged and Induced‐senescent groups impacted overall tissue composition over the course of culture. At day 14, we found significant decreases in DNA content in aged, RAD, and DOX explants compared to the young group (Figure 5g). While reduced DNA content is typically attributed to diminished cell numbers, we did not find a corresponding change in cell viability. However, our DNA content assay is a quantification of double‐stranded DNA (Singer et al., 1997), and therefore it's possible this reflects senescence‐associated DNA damage in induced groups later in culture. Interestingly, there were no differences in collagen content over the course of culture (Figure 5h) despite significant decreases in collagen gene expression and proline incorporation. We suspect this may be due to either differential protein degradation processes (MMPs) or the relative difference in turnover rates between GAGs and collagen, as GAG is expected to turnover much more rapidly (Choi et al., 2020). Supporting this, we did find significant reductions in both total GAG (Figure 5i) and water content (data not shown) at day 14 in aged and induced‐senescent groups. While we did not repeat baseline tissue compositional measurements in this study (Connizzo et al., 2020), it's worth noting that ionizing irradiation could cause radiolysis and initial matrix damage in RAD tendons that could affect results. However, we did not find any differential results with RAD compared to DOX and aged groups to suggest this was the case. Overall, our multi‐scale assessment demonstrates that both aged and induced‐senescent tendons exhibit a decreased capacity to synthesize new ECM in response to mechanical unloading.

### 3.4 Induced‐senescent explants show sustained matrix degradation and inflammatory responses

**FIGURE 6:**
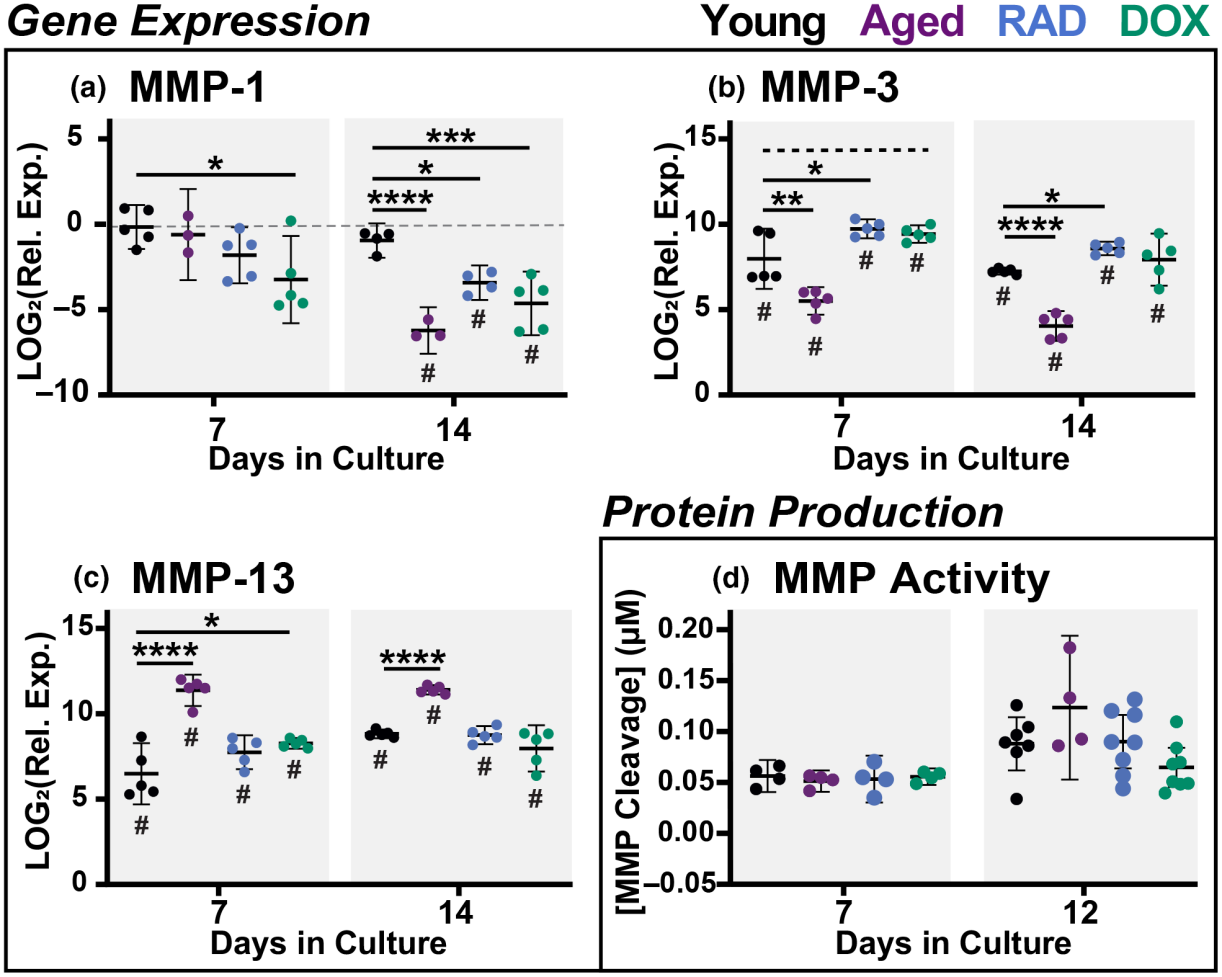
Matrix Degradation. Gene expression of (a) MMP‐1, (b) MMP‐3, and (c) MMP‐13 at days 7 and 14. Gene expression data presented as relative expression to D0. (d) Generic MMP protein activity in culture medium at days 7 and 12. Significant statistical comparisons to young explants are shown with **** for *p* < 0.0001, *** for *p* < 0.001, ** for *p* < 0.01, and * for *p* < 0.05. Trends (*p* < 0.1) are shown with dashed lines. # indicates significant differences from day 0 values (*p* < 0.05).

We also assessed ECM breakdown via production of MMPs and secreted inflammatory proteins for young, aged, and induced‐senescent tissues. While ECM degradation is an essential part of routine matrix turnover, extensive breakdown resulting from highly inflammatory states can compromise tissue integrity. Expression of MMP‐1 was decreased in aged and induced‐senescent tissues compared to young tissues, with differences that become more pronounced by day 14 (Figure 6a). MMP‐1 is specifically involved in the breakdown of interstitial collagens, such as type 1 and type 3 collagen, suggesting both reduced collagen breakdown and potentially a profibrotic healing response (Singh et al., 2023). Interestingly, Brauer et al. (2022) previously reported increased MMP‐1 expression with DNA damage‐mediated senescence but decreased MMP‐1 expression in DNA damage‐independent senescence compared to controls, suggesting induction‐specific expression of MMP‐1. MMP‐3, responsible for proteoglycan cleavage (Cui et al., 2017), was significantly upregulated in all groups throughout culture (Figure 6b,c). While aged tendons had reduced expression of MMP‐3 compared to young, induced‐senescent explants had increased MMP‐3 expression at days 7 (RAD & DOX) and 14 (DOX only) (Figure 6b). Elevated expression of MMP‐3 could signify increased proteoglycan and GAG breakdown, supporting our earlier findings of reduced GAG content in aged and induced‐senescent tendons. MMP‐13, a collagenase, was also upregulated in all groups throughout culture. Both aged and DOX tendons had significantly higher MMP‐13 expression than young tissues at day 7 (Figure 6c). By day 14, only aged tissues had increased MMP‐13 expression compared to young. This data suggests sustained increases in collagen degradation in aged tendons that may be attributed to senescent cell populations.

We also assessed MMP protein activity and inflammatory protein secretion at 2, 7, and 12‐days after the initiation of the stress deprivation injury (Figure 6d and Figure S4). Under stress deprivation, MMP activity increases consistently throughout the culture period (Aggouras et al., 2024). In aged and induced‐senescent tissues, MMP activity was on par with young tissues, with no significant differences between groups (Figure 6d). It's important to note here that this assay is reflective of nine different types of MMPs, and therefore we did not have individual MMP protein activity in this study. Given our observation that senescence alters expression of different MMPs in distinct ways, future work will look at the production of individual MMPs, as well as tissue inhibitors of MMPs (TIMPs), to provide more nuanced insights on tissue degradation. Techniques such as in situ zymography or immunohistochemistry for specific MMPs will also yield more localized information on MMP activity in tissue sections.

Similarly, the production of IL‐6, MIP‐1α, MCP‐1, IL‐13, and TNFα increased throughout culture for all groups, with little significant differences between groups (Figure S4). Therefore, the magnitude of the induced‐senescent response is on par with that of a mechanical injury and sustained throughout the culture period. This was contrary to our initial hypothesis that induced‐senescent tendon explants would display substantial increases in tissue breakdown due to the presence of the SASP phenotype. However, because we are simultaneously assessing compounding the effects of senescence induction and a mechanical unloading injury (stress deprivation), the interpretation of individual biomarkers is more complex.

### 3.5 Induced‐senescent phenotype and disrupted ECM homeostasis are independent of mechanical factors

It is clear that mechanical unloading alone has substantial impact on cell health at late stages of culture. Specifically, we observe declines in tissue viability and cell proliferation, accompanied by increased expression of injury and apoptosis markers, increased MMP activity, and secretion of inflammatory factors. In alignment with previous work (Aggouras et al., 2024; Egerbacher et al., 2008), these biomarkers support a highly‐inflammatory and injurious response to mechanical unloading causing apoptosis and cellular degradation in young tissues. Surprisingly, we also found significantly increased SA‐β‐Gal, p16, and p21 in young tissues at day 7 compared to freshly harvested tissues. It is possible that the mechanical unloading could induce some cells to cellular senescence via an injury‐initiated mechanism. Previous work has reported in vivo senescence markers (reduced cell division, p19, p53) in patellar tendons following injections of Botox to induce mechanical stress‐deprivation (Chen et al., 2021). Many other groups are also interested in this idea of injury‐initiated cellular senescence, particularly in the context of wound healing (Samdavid Thanapaul et al., 2022). However, there is still much debate about the interplay of induced‐senescent cells and tissue healing, particularly the permanent nature of these observed induced‐senescent phenotypes (Chu et al., 2020). Additionally, we did not find cell cycle arrest in concert with other senescence‐associated changes leading to questions regarding the distinctions between senescence, quiescence, and early apoptosis. Regardless, stress deprivation alone clearly has pronounced effects on the expression of multiple biomarkers of cell health, making it difficult to tease out the phenotype of senescence induction from that of chronic mechanical injury.

To minimize this confounding variable, we also performed preliminary senescence induction studies under physiological levels of mechanical loading (Figure S5). We previously found that low levels of cyclic tensile strain best maintain native tendon physiology in explant culture (Aggouras et al., 2024). Using an identical experimental setup to previously published work, we explored the effects of senescence induction with DOX under 3% cyclic tensile strain (Figure S5). Cyclically‐loaded DOX tissues demonstrate show similar trends to unloaded tissues with reduced metabolic activity, proliferation, p16, lmb1, and collagen 1 expression while showing increased p53, p21, casp3, IL‐6, decorin, fibromodulin, and MMP‐3 expression (Figure S5). Interestingly, even under tensile loading, no SASP phenotype was found at the protein level with no differences in MMP activity or inflammatory protein production at day 7 between young and DOX‐treated tissues. It is possible that SASP is not a defining feature of senescence in tissue with low relative metabolic function, such as tendon, that tendon‐specific SASP is characterized by markers that were not measured here, or the presence of a SASP is induction‐specific (Brauer et al., 2022). It's also possible a differential SASP would take longer than 7 days to develop. These data further support our initial findings and suggest that while senescence‐related effects are likely independent of mechanical stimulus, there is complex interplay between mechanical state and common senescence‐associated markers.

## 4 CONCLUSIONS

Looking at all the data together, we see that compared to young tendons under identical mechanical stimuli, induced‐senescent tendons exhibit reduced matrix synthesis, thus shifting the remodeling balance towards degradation over production. Interestingly, changes in ECM remodeling found here were similar between the naturally aged and induced‐senescent tendons, suggesting that senescence likely contributes to divergent remodeling outcomes in aged tissues. In some cases, the remodeling response appears graded, with more severe changes in the induced‐senescent groups than in aged tissues, possibly due to the fewer number of senescent cells in naturally aged than induced‐senescent tissues. In other cases, the aged tissue response is no different from induced‐senescent tissues, suggesting that the presence of a small number of induced‐senescent cells is enough to compromise bulk tissue responses. Regardless, induced‐senescent and aged tendons have a reduced capacity to respond and adapt to altered mechanical environments, likely contributing to the increased prevalence of tendon injuries and poor healing outcomes observed in aging populations.

To our knowledge, this is one of the first studies to induce in vitro cellular senescence within the native ECM. This novel senescent explant model allows us to directly assess the contributions of senescent cell populations to altered extracellular matrix remodeling. Importantly, we are able to compare the response of senescent tenocytes in 2D cell culture with those remaining in their native microenvironment enabling a more physiologically relevant understanding of the functional consequences of senescence in tendon health. We demonstrate the utility of this model in exploring altered processes of ECM remodeling by investigating how the ECM turnover is disrupted in both naturally aged and induced‐senescent tissues following a mechanical unloading injury. From a clinical perspective, this work suggests that senolytic therapies that selectively eliminate induced‐senescent cell populations may be a promising strategy for preventing age‐related tendon degeneration (Chaib et al., 2022). In the future, this model will give us the opportunity to screen potential treatment strategies in a physiologically relevant context and assess the therapeutic ability of senescence‐targeting drugs to rescue homeostatic ECM remodeling.

## AUTHOR CONTRIBUTIONS

EJ Stowe has contributed to all aspects of this study, including research design, data acquisition, interpretation/analysis of data, and drafting/revision of manuscript. MR Keller has contributed to data acquisition, interpretation/analysis of data, and drafting/revision of manuscript. BK Connizzo has contributed significantly to research design, interpretation/analysis of data, and drafting/revision of the manuscript. All authors have read and approved the final submitted manuscript.

## FUNDING INFORMATION

This study was supported by NIH/NIA K99/R00‐AG063896 and NSF GFRP (Stowe).

## CONFLICT OF INTEREST STATEMENT

The authors declare that the research was conducted in the absence of any commercial or financial relationships that could be construed as a potential conflict of interest.

## Supporting information


Appendix S1.

## Data Availability

The data that support the findings in this study are available from the corresponding author upon reasonable request.
