# Supplementary material for: Cellular senescence impairs tendon extracellular matrix remodeling in response to mechanical unloading
Source: Aging Cell. 2024 Jul 22;23(11):e14278. doi: 10.1111/acel.14278 (PMC11561669; doi:10.1111/acel.14278)
Supplement: Supplementary file 1 — Appendix S1. [file ACEL-23-e14278-s001.pdf]

Cellular Senescence Impairs Tendon Extracellular Matrix Remodeling in Response to Mechanical Unloading

Supporting Information

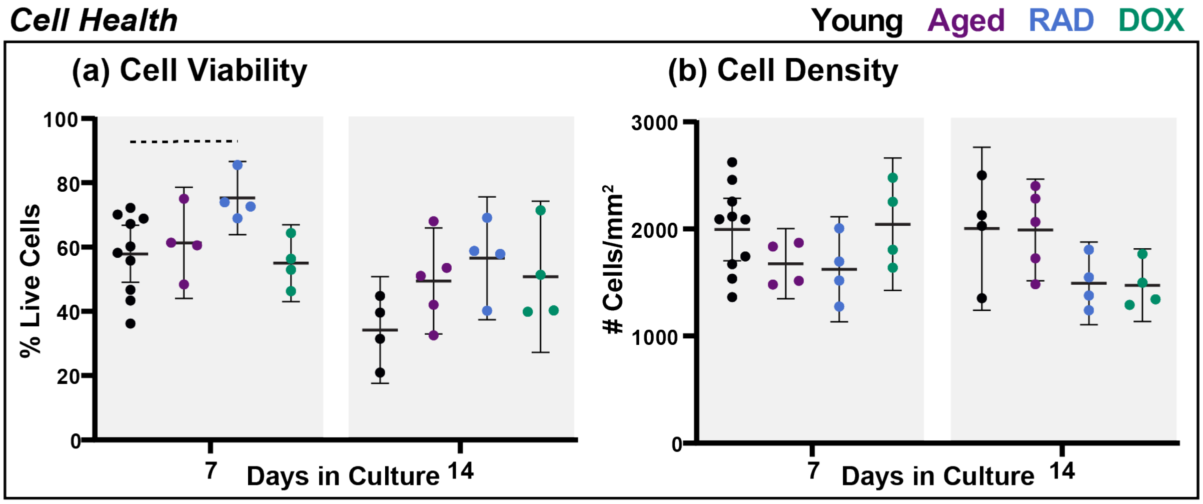

Supporting Figure S1: (a) Explant cell viability and (b) cell density quantified from confocal z-stacks stained for viability (live/dead). Significant statistical comparisons to young explants are shown with **** for p<0.0001, *** for p<0.001, ** for p<0.01, and * for p<0.05 (solid lines). Trends (p<0.1) are shown with dashed lines.

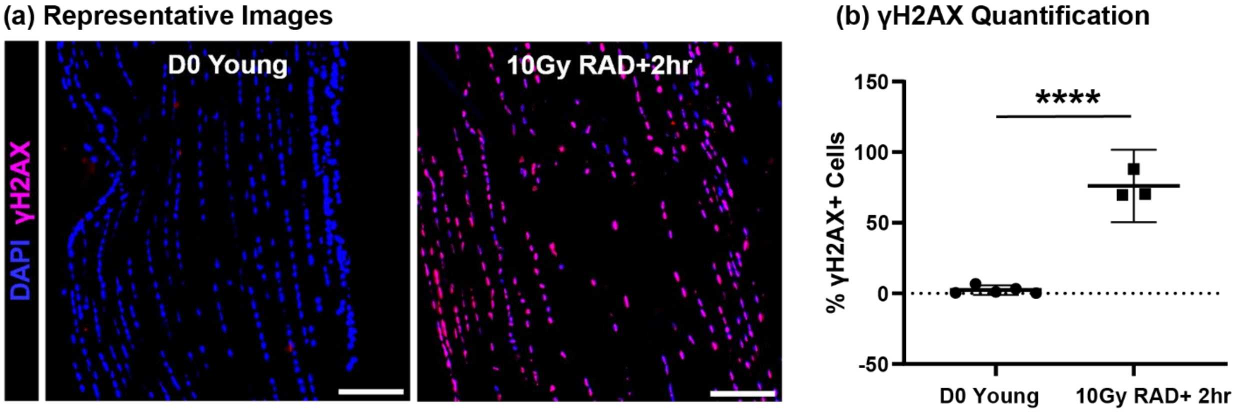

Supporting Figure S2: (a) Representative images and (b) associated quantification of γH2AX DNA damage positive control. Young tissue explants were treated with 10Gy irradiation and left in culture for 2-hours before sample takedown. DAPI shown in blue and γH2AX co-staining in magenta. Scale bars are 100µm. Significant statistical comparisons are shown with **** for p<0.001, *** for p<0.001, ** for p<0.01, and * for p<0.05.

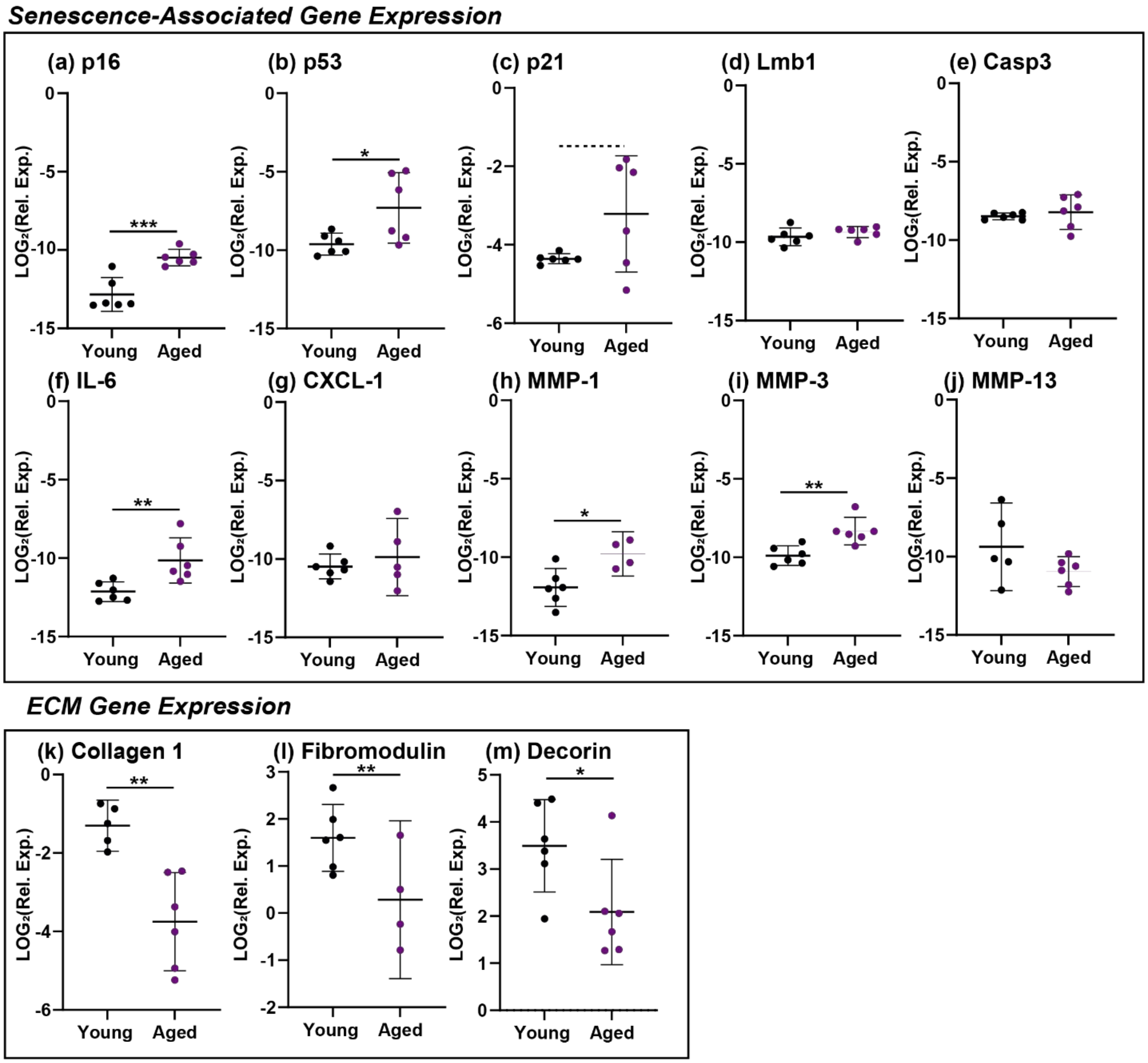

Supporting Figure S3: Baseline expression (day 0) of senescence genes (a) p16, (b) p53, (c) p21, (d) Lmb1, (e) Casp3, (f) IL-6, (g) CXCL-1, (h) MMP-1, (i) MMP-3, (j) MMP-13 and ECM genes (k) collagen 1, (l) fibromodulin, and (m) decorin. Significant statistical comparisons between young and aged freshly harvested tissues are shown with **** for $p<0.0001$, *** for $p<0.001$, ** for $p<0.01$, and * for $p<0.05$ (solid lines). Trends ($p<0.1$) are shown with dashed lines.

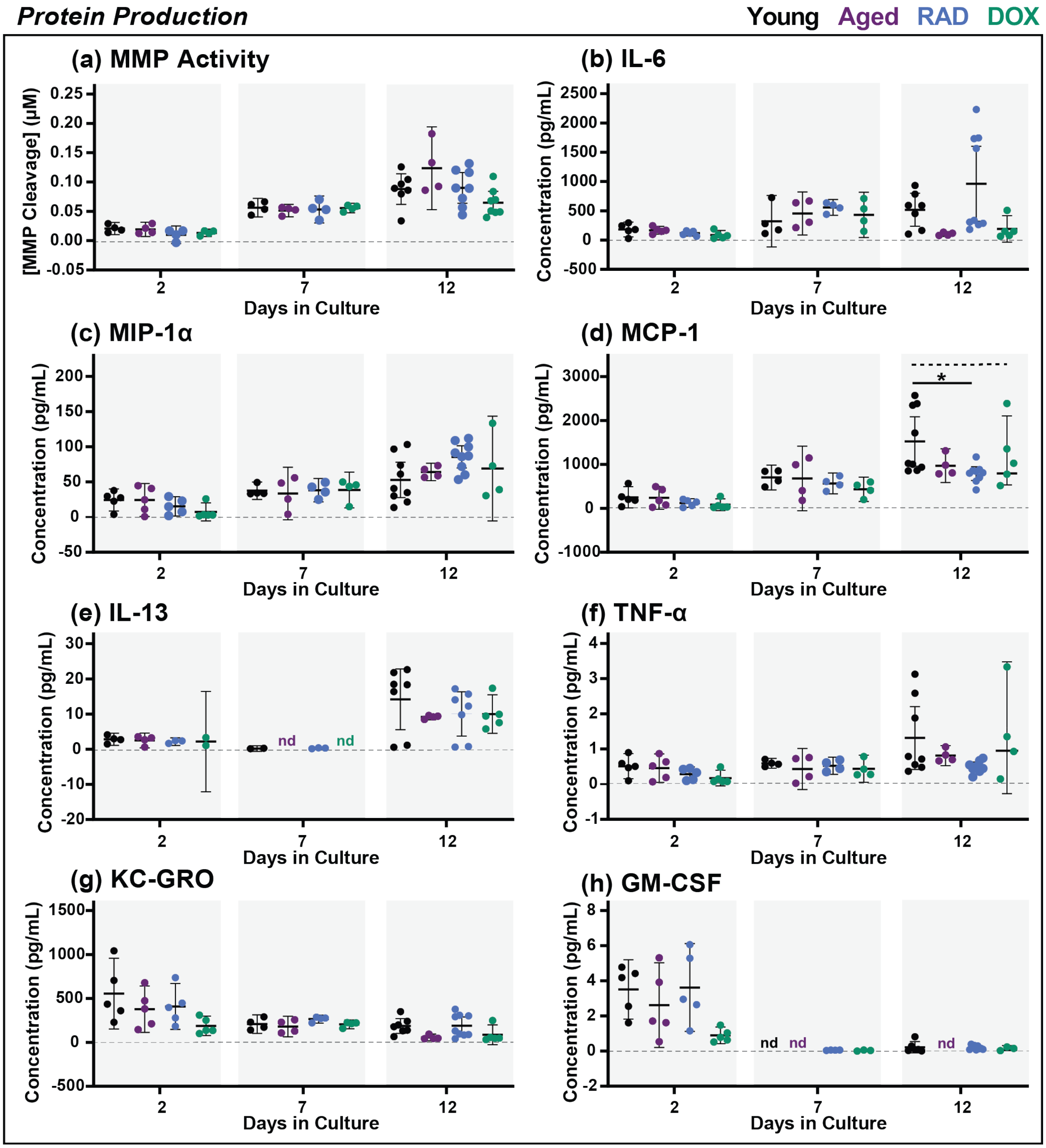

Supporting Figure S4: (a) Generic MMP protein activity and (b-h) concentration of secreted inflammatory proteins in culture media at days 2,7, and 12. "nd" is listed where concentrations were below the detection limit of the assay and therefore not detected. Significant statistical comparisons to young explants are shown with **** for p<0.0001, *** for p<0.001, ** for p<0.01, and * for p<0.05 (solid lines). Trends (p<0.1) are shown with dashed lines.

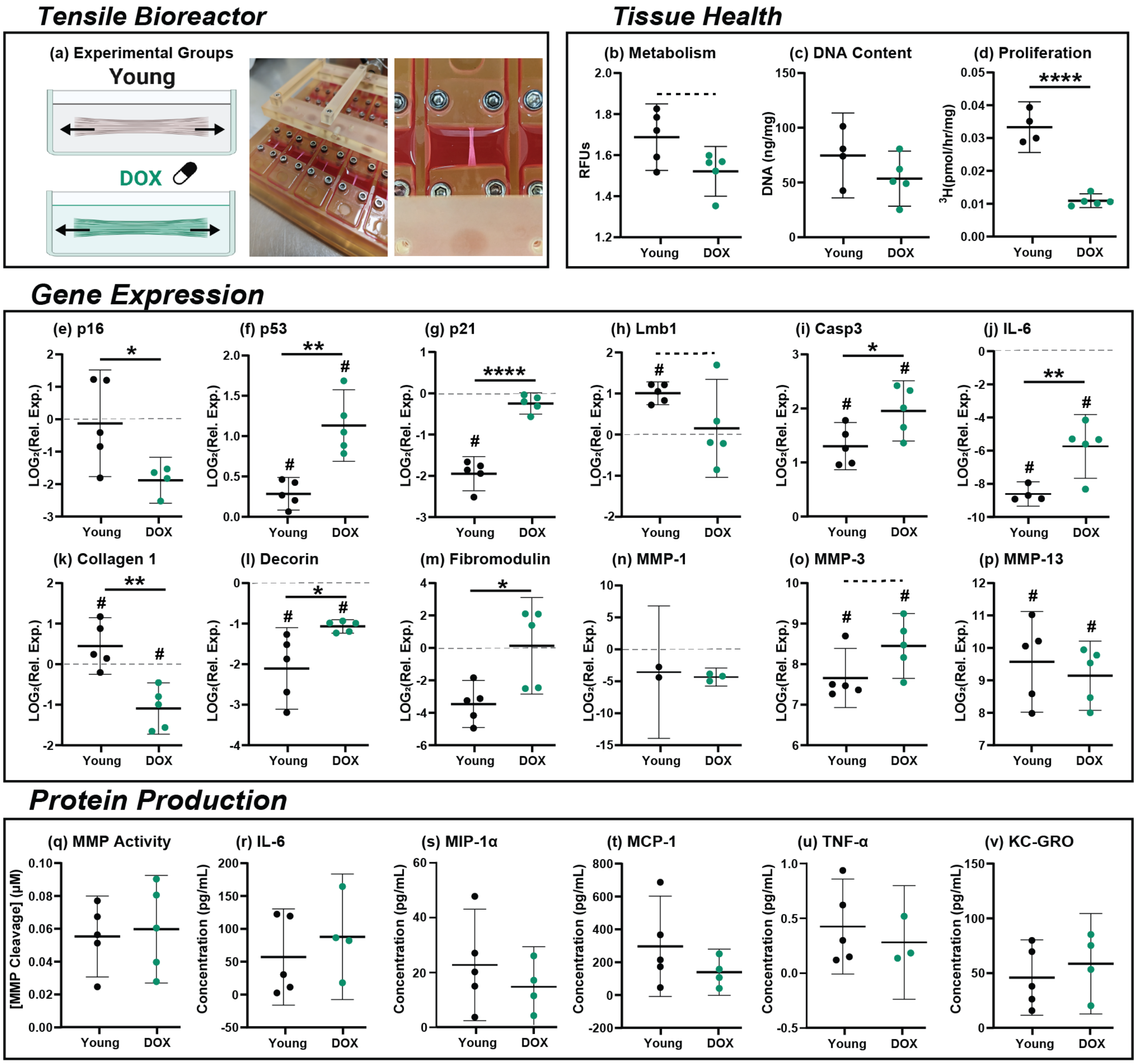

Supporting Figure S5: Senescence induction with DOX treatment was performed under cyclic tensile strain using a custom built tensile-loading bioreactor to remove the confounding effects of mechanical unloading. (a) Experimental groups and bioreactor photos. Young and DOX tendon explants were assessed at day 7 for (b) metabolism (c) DNA content, and (d) proliferation. Day 7 gene expression of (e) p16, (f) p53, (g) p21, (h) Lmb1, (i) Casp3, (j) IL-6, (k) collagen 1, (l) decorin, (m) fibromodulin, (n) MMP-1, (o) MMP-3, and (p) MMP-13 is presented relative to D0 baseline values. (q) MMP activity and concentration of secreted proteins (r-v) in culture medium was assessed at day 7. Significant statistical comparisons between young and DOX tissues are shown with **** for $p<0.0001$, *** for $p<0.001$, ** for $p<0.01$, and * for $p<0.05$ (solid lines). Trends ($p<0.1$) are shown with dashed lines. # indicates significant differences from day 0 values ($p<0.05$).

Supporting Table S1. Murine gene names and primer forward/reverse sequences used for quantitative gene expression analysis.

Target	Gene Name	Forward (5'-> 3')	Reverse (5'-> 3')
Beta Actin	*Actb* [1]	GGCTGTATTCCCCTCCATCG	CCAGTTGGTAACAATGCCATGT
p16^{ink4a}	*Cdkn2a* [2]	CGGTCGTACCCCGATTCAG	GCACCGTAGTTGAGCAGAAGAG
p53	*Tp53* [2]	GTACCACCATCCACTACAACTACAT	CAGGACAGGCACAAACACG
p21	*Cdkn1a*[3]	CCTGGTGATGTCCGACCTG	CCATGAGCGCATCGCAATC
Lamin B1	*Lmb1* [4]	GGGAAGTTTATTCGCTTGAAGA	ATCTCCCAGCCTCCCATT
Caspase-3	*Casp3* [5]	ATGGGAGCAAGTCAGTGGAC	CGTACCAGAGCGAGATGACA
IL-6	*Il6* [2]	TAGCTACCTGGAGTACATGAAGAACA	TGGTCCTTAGCCACTCCTTCTG
MMP-1	*Mmp1* [6]	TCAACCAGGCCAAGGTATTG	ATGAGCAGCCACGAGAAATAG
MMP-3	*Mmp3* [5]	ACATGGAGACTTTGTCCCTTTTG	TTGGCTGAGTGGTAGAGTCCC
MMP-13	*Mmp13* [5]	TCAGTCTCTTCACCTCTTTTGGGAATCC	TCAGTTTCTTTATGGTCCAGGCGATG
Collagen 1	*Col1a1* [2]	GACATGTTCAGCTTTGTGGACCTC	GGGACCCTTAGGCCATTGTGTA
Decorin	*Dcn* [2]	CTATGTGCCCCTACCGATGC	CAGAACACTGCACCACTCGAAG
Fibromodulin	*Fmod* [2]	CTCCAACCCAAGGAGACCAG	GGATCCACCAGTGAGAGTCTTC

[1] Veres-Székely, A., Pap, D., Sziksz, E., Jávorszky, E., Rokonay, R., Lippai, R., Tory, K., Fekete, A., Tulassay, T., Szabó, A. J., and Vannay, Á., 2017, “Selective Measurement of α Smooth Muscle Actin: Why β-Actin Can Not Be Used as a Housekeeping Gene When Tissue Fibrosis Occurs,” BMC Mol Biol, **18**(1), p. 12.

[2] Connizzo, B. K., Piet, J. M., Shefelbine, S. J., and Grodzinsky, A. J., 2020, “Age-Associated Changes in the Response of Tendon Explants to Stress Deprivation Is Sex-Dependent,” Connect Tissue Res, **61**(1), pp. 48–62.

[3] Chen, J., Sun, Z.-H., Chen, L.-Y., Xu, F., Zhao, Y.-P., Li, G.-Q., Tang, M., Li, Y., Zheng, Q.-Y., Wang, S.-F., Yang, X.-H., Wu, Y.-Z., and Xu, G.-L., 2020, “C5aR Deficiency Attenuates the Breast Cancer Development via the P38/P21 Axis,” Aging (Albany NY), **12**(14), pp. 14285–14299.

[4] Freund, A., Laberge, R.-M., Demaria, M., and Campisi, J., 2012, “Lamin B1 Loss Is a Senescence-Associated Biomarker,” Mol Biol Cell, **23**(11), pp. 2066–2075.

[5] Connizzo, B. K., and Grodzinsky, A. J., 2018, “Release of Pro-Inflammatory Cytokines from Muscle and Bone Causes Tenocyte Death in a Novel Rotator Cuff in Vitro Explant Culture Model,” Connect Tissue Res, **59**(5), pp. 423–436.

[6] Wei, X., and Shao, X., 2018, “Nobiletin Alleviates Endometriosis via Down-Regulating NF-κB Activity in Endometriosis Mouse Model,” Biosci Rep, **38**(3), p. BSR20180470.

Supporting Table S2. Figure 2 statistics. One-way ANOVA with Tukey's multiple comparisons for all groups. ANOVA was significant if multiple comparisons are shown.

**SA-β-Gal+ Cells**		
Tukey's multiple comparisons test	Summary	Adjusted P Value
**Young vs. Aged**	***	**0.0008**
**Young vs. DOX**	***	**0.0002**
**Young vs. RAD**	***	**0.0001**
Aged vs. DOX	ns	0.8964
Aged vs. RAD	ns	0.6655
DOX vs. RAD	ns	0.9674
**γH2AX+ Cells**		
Tukey's multiple comparisons test	Summary	Adjusted P Value
**Young vs. Aged**	*	**0.0162**
**Young vs. DOX**	****	**<0.0001**
**Young vs. RAD**	****	**<0.0001**
Aged vs. DOX	****	<0.0001
Aged vs. RAD	***	0.0005
DOX vs. RAD	**	0.0013
**Double+ Cells**		
Tukey's multiple comparisons test	Summary	Adjusted P Value
**Young vs. Aged**	*	**0.0241**
**Young vs. DOX**	****	**<0.0001**
**Young vs. RAD**	****	**<0.0001**
Aged vs. DOX	***	0.0004
Aged vs. RAD	*	0.0195
DOX vs. RAD	ns	0.1636
**# γH2AX Foci**		
Tukey's multiple comparisons test	Summary	Adjusted P Value
**Young vs. Aged**	**ns**	**0.5962**
**Young vs. DOX**	****	**<0.0001**
**Young vs. RAD**	***	**0.0005**
Aged vs. DOX	***	0.0002
Aged vs. RAD	**	0.0064
DOX vs. RAD	ns	0.1793
**Proliferation**		
Tukey's multiple comparisons test	Summary	Adjusted P Value
**Young vs. Aged**	**	**0.0018**
**Young vs. DOX**	**	**0.0028**
**Young vs. RAD**	**ns**	**0.0855**
Aged vs. DOX	ns	0.997
Aged vs. RAD	ns	0.208

DOX vs. RAD	ns	0.2874
**Cell Metabolism**		
Tukey's multiple comparisons test	Summary	Adjusted P Value
**Young vs. Aged**	**ns**	**0.9825**
**Young vs. DOX**	**ns**	**0.1356**
**Young vs. RAD**	**ns**	**0.2859**
Aged vs. DOX	ns	0.3375
Aged vs. RAD	ns	0.5642
DOX vs. RAD	ns	0.9699

Supporting Table S3. Figure 3 statistics. One-way ANOVA at each timepoint with Tukey's multiple comparisons for all groups. ANOVA was significant if multiple comparisons are shown. Day 0 comparisons not shown.

**Proliferation- Day 1**		
Tukey's multiple comparisons test	Summary	Adjusted P Value
**Young vs. RAD**	**ns**	**0.1337**
**Young vs. DOX**	**	**0.0068**
RAD vs. DOX	ns	0.2482
**Proliferation- Day 3**		
Tukey's multiple comparisons test	Summary	Adjusted P Value
**Young vs. RAD**	***	**0.0002**
**Young vs. DOX**	**	**0.0013**
RAD vs. DOX	ns	0.5428
**Proliferation- Day 5**		
Tukey's multiple comparisons test	Summary	Adjusted P Value
**Young vs. RAD**	****	**<0.0001**
**Young vs. DOX**	****	**<0.0001**
RAD vs. DOX	ns	0.5148
**Proliferation- Day 7**		
Tukey's multiple comparisons test	Summary	Adjusted P Value
**Young vs. Aged**	**	**0.0058**
**Young vs. RAD**	****	**<0.0001**
**Young vs. DOX**	****	**<0.0001**
Aged vs. RAD	****	<0.0001
Aged vs. DOX	****	<0.0001
RAD vs. DOX	ns	0.9706
**Proliferation- Day 10**		
Tukey's multiple comparisons test	Summary	Adjusted P Value
**Young vs. RAD**	***	**0.0002**
**Young vs. DOX**	***	**0.0005**
RAD vs. DOX	ns	0.8295

**Proliferation- Day 14**		
Tukey's multiple comparisons test	Summary	Adjusted P Value
**Young vs. RAD**	*******	**0.0003**
**Young vs. DOX**	******	**0.0035**
RAD vs. DOX	ns	0.4537
**Apoptosis Resistance- Day 7**		
Tukey's multiple comparisons test	Summary	Adjusted P Value
**Young vs. Aged**	**ns**	**0.8996**
**Young vs. RAD**	*****	**0.0102**
**Young vs. DOX**	*****	**0.0429**
Aged vs. RAD	**	0.0025
Aged vs. DOX	*	0.0109
RAD vs. DOX	ns	0.8876
**p16 Expression- Day 7**		
Tukey's multiple comparisons test	Summary	Adjusted P Value
**Young vs. Aged**	*****	**0.0158**
**Young vs. RAD**	*****	**0.0484**
**Young vs. DOX**	**ns**	**0.2015**
Aged vs. RAD	ns	0.9404
Aged vs. DOX	ns	0.5377
RAD vs. DOX	ns	0.8545
**p16 Expression- Day 14**		
Tukey's multiple comparisons test	Summary	Adjusted P Value
**Young vs. Aged**	*****	**0.0112**
**Young vs. RAD**	*******	**0.0002**
**Young vs. DOX**	******	**0.0094**
Aged vs. RAD	ns	0.2229
Aged vs. DOX	ns	0.9997
RAD vs. DOX	ns	0.2556
**p53 Expression- Day 7**		
Tukey's multiple comparisons test	Summary	Adjusted P Value
**Young vs. Aged**	**ns**	**0.8357**
**Young vs. RAD**	**ns**	**0.1669**
**Young vs. DOX**	**ns**	**0.2338**
Aged vs. RAD	*	0.0359
Aged vs. DOX	ns	0.0536
RAD vs. DOX	ns	0.9967
**p53 Expression- Day 14**		
Tukey's multiple comparisons test	Summary	Adjusted P Value
**Young vs. Aged**	********	**<0.0001**
**Young vs. RAD**	**ns**	**0.9958**

**Young vs. DOX**	**ns**	**0.2743**
Aged vs. RAD	****	<0.0001
Aged vs. DOX	****	<0.0001
RAD vs. DOX	ns	0.3772
**p21 Expression- Day 7**		
Tukey's multiple comparisons test	Summary	Adjusted P Value
**Young vs. Aged**	**ns**	**0.9986**
**Young vs. RAD**	**	**0.0015**
**Young vs. DOX**	**	**0.0027**
Aged vs. RAD	**	0.0021
Aged vs. DOX	**	0.0037
RAD vs. DOX	ns	0.9912
**p21 Expression- Day 14**		
Tukey's multiple comparisons test	Summary	Adjusted P Value
**Young vs. Aged**	****	**<0.0001**
**Young vs. RAD**	**ns**	**0.2949**
**Young vs. DOX**	**ns**	**0.5383**
Aged vs. RAD	****	<0.0001
Aged vs. DOX	****	<0.0001
RAD vs. DOX	*	0.026
**Lmb1 Expression- Day 7**		
ANOVA summary		
F	0.1927	
P value	0.8998	
P value summary	ns	
Significant diff. among means (P < 0.05)?	No	
R squared		
**Lmb1 Expression- Day 14**		
**Tukey's multiple comparisons test**	**Summary**	**Adjusted P Value**
**Young vs. Aged**	**ns**	**0.5017**
**Young vs. RAD**	*	**0.031**
**Young vs. DOX**	**ns**	**0.4819**
Aged vs. RAD	ns	0.3654
Aged vs. DOX	ns	>0.9999
RAD vs. DOX	ns	0.3827
**Casp3 Expression- Day 7**		
Tukey's multiple comparisons test	Summary	Adjusted P Value
**Young vs. Aged**	*	**0.0358**
**Young vs. RAD**	**	**0.0056**
**Young vs. DOX**	**	**0.0069**
Aged vs. RAD	ns	0.7939

Aged vs. DOX	ns	0.8435
RAD vs. DOX	ns	0.9996
**Casp3 Expression- Day 14**		
ANOVA summary	2.852	
F	0.0702	
P value	ns	
P value summary	No	
Significant diff. among means (P < 0.05)?	0.3484	
R squared	2.852	
**IL-6 Expression- Day 7**		
Tukey's multiple comparisons test	Summary	Adjusted P Value
**Young vs. Aged**	**ns**	**0.1161**
**Young vs. RAD**	**	**0.004**
**Young vs. DOX**	**ns**	**0.1343**
Aged vs. RAD	****	<0.0001
Aged vs. DOX	**	0.0012
RAD vs. DOX	ns	0.3098
**IL-6 Expression- Day 14**		
Tukey's multiple comparisons test	Summary	Adjusted P Value
**Young vs. Aged**	****	**<0.0001**
**Young vs. RAD**	**ns**	**0.9154**
**Young vs. DOX**	**ns**	**>0.9999**
Aged vs. RAD	****	<0.0001
Aged vs. DOX	****	<0.0001
RAD vs. DOX	ns	0.9239

Supporting Table S4. Figure 4 statistics. One-way ANOVA or unpaired t-test at each timepoint. Day 0 comparisons not shown.

**yH2AX Day 0**		
Unpaired t test		
P value	0.5357	
P value summary	ns	
Significantly different (P < 0.05)?	No	
One- or two-tailed P value?	Two-tailed	
**yH2AX Day 7**		
ANOVA summary		
F	0.2033	
P value	0.8925	
P value summary	ns	
Significant diff. among means (P < 0.05)?	No	
R squared	0.03908	

**SABG Day 0**		
Unpaired t test		
P value	0.9741	
P value summary	ns	
Significantly different (P < 0.05)?	No	
One- or two-tailed P value?	Two-tailed	
**SABG Day 7**		
ANOVA summary		
F	1.349	
P value	0.296	
P value summary	ns	
Significant diff. among means (P < 0.05)?	No	
R squared	0.2125	
**p21 Day 0**		
Unpaired t test		
P value	0.0292	
P value summary	*	
Significantly different (P < 0.05)?	Yes	
One- or two-tailed P value?	Two-tailed	
**p21 Day 7**		
ANOVA summary		
F	2.258	
P value	0.13	
P value summary	ns	
Significant diff. among means (P < 0.05)?	No	
R squared	0.3426	

Supporting Table S5. Figure 5 statistics. One-way ANOVA at each timepoint with Tukey's multiple comparisons for all groups. ANOVA was significant if multiple comparisons are shown. Day 0 comparisons not shown.

**Collagen 1 Gene Expression - Day 7**		
Tukey's multiple comparisons test	Summary	Adjusted P Value
**Young vs. Aged**	**	**0.0076**
**Young vs. RAD**	**ns**	**0.9986**
**Young vs. DOX**	**ns**	**0.8761**
Aged vs. RAD	*	0.0104
Aged vs. DOX	*	0.0341
RAD vs. DOX	ns	0.9332
**Collagen 1 Gene Expression - Day 14**		
Tukey's multiple comparisons test	Summary	Adjusted P Value
**Young vs. Aged**	**ns**	**0.6382**

**Young vs. RAD**	*	**0.0134**
**Young vs. DOX**	*	**0.0435**
Aged vs. RAD	ns	0.1319
Aged vs. DOX	ns	0.3375
RAD vs. DOX	ns	0.9323
**Decorin Gene Expression - Day 7**		
Tukey's multiple comparisons test	Summary	Adjusted P Value
**Young vs. Aged**	***	**0.0004**
**Young vs. RAD**	*	**0.0239**
**Young vs. DOX**	**Trend**	**0.0897**
Aged vs. RAD	ns	0.2327
Aged vs. DOX	Trend	0.0706
RAD vs. DOX	ns	0.8997
**Decorin Gene Expression - Day 14**		
Tukey's multiple comparisons test	Summary	Adjusted P Value
**Young vs. Aged**	**ns**	**0.999**
**Young vs. RAD**	*	**0.0383**
**Young vs. DOX**	*	**0.0418**
Aged vs. RAD	*	0.0499
Aged vs. DOX	Trend	0.0544
RAD vs. DOX	ns	>0.9999
**Fibromodulin Gene Expression - Day 7**		
Tukey's multiple comparisons test	Summary	Adjusted P Value
**Young vs. Aged**	*	**0.0343**
**Young vs. RAD**	**Trend**	**0.0987**
**Young vs. DOX**	**ns**	**0.2198**
Aged vs. RAD	ns	0.9424
Aged vs. DOX	ns	0.7346
RAD vs. DOX	ns	0.9645
**Fibromodulin Gene Expression - Day 14**		
Tukey's multiple comparisons test	Summary	Adjusted P Value
**Young vs. Aged**	****	**<0.0001**
**Young vs. RAD**	***	**0.0003**
**Young vs. DOX**	***	**0.0004**
Aged vs. RAD	ns	0.5166
Aged vs. DOX	ns	0.3844
RAD vs. DOX	ns	0.9949
**Metabolism - Day 7**		
Tukey's multiple comparisons test	Summary	Adjusted P Value
**Young vs. Aged**	**	**0.001**
**Young vs. RAD**	****	**<0.0001**

**Young vs. DOX**	****	**<0.0001**
Aged vs. RAD	ns	0.6125
Aged vs. DOX	Trend	0.06
RAD vs. DOX	ns	0.6043
**Metabolism - Day 14**		
Tukey's multiple comparisons test	Summary	Adjusted P Value
**Young vs. Aged**	*	**0.0151**
**Young vs. RAD**	***	**0.0003**
**Young vs. DOX**	**	**0.001**
Aged vs. RAD	ns	0.2182
Aged vs. DOX	ns	0.5657
RAD vs. DOX	ns	0.8905
**Total Protein Synthesis - Day 7**		
Tukey's multiple comparisons test	Summary	Adjusted P Value
**Young vs. Aged**	**Trend**	**0.0634**
**Young vs. RAD**	**	**0.0056**
**Young vs. DOX**	**	**0.0023**
Aged vs. RAD	ns	0.6247
Aged vs. DOX	ns	0.3711
RAD vs. DOX	ns	0.9686
**Total Protein Synthesis - Day 14**		
Tukey's multiple comparisons test	Summary	Adjusted P Value
**Young vs. Aged**	***	**0.0007**
**Young vs. RAD**	****	**<0.0001**
**Young vs. DOX**	****	**<0.0001**
Aged vs. RAD	ns	0.148
Aged vs. DOX	ns	0.22
RAD vs. DOX	ns	0.9949
**sGAG Synthesis - Day 7**		
Tukey's multiple comparisons test	Summary	Adjusted P Value
**Young vs. Aged**	*	**0.0102**
**Young vs. RAD**	**	**0.002**
**Young vs. DOX**	***	**0.0009**
Aged vs. RAD	ns	0.8494
Aged vs. DOX	ns	0.6506
RAD vs. DOX	ns	0.9826
**sGAG Synthesis - Day 14**		
Tukey's multiple comparisons test	Summary	Adjusted P Value
**Young vs. Aged**	**	**0.0011**
**Young vs. RAD**	****	**<0.0001**
**Young vs. DOX**	***	**0.0004**

Aged vs. RAD	ns	0.4675
Aged vs. DOX	ns	0.9404
RAD vs. DOX	ns	0.7947
**DNA Content - Day 7**		
ANOVA summary		
F	1.24	
P value	0.328	
P value summary	ns	
Significant diff. among means (P < 0.05)?	No	
**DNA Content - Day 14**		
Tukey's multiple comparisons test	Summary	Adjusted P Value
**Young vs. Aged**	********	**<0.0001**
**Young vs. RAD**	********	**<0.0001**
**Young vs. DOX**	*******	**0.0001**
Aged vs. RAD	ns	0.884
Aged vs. DOX	ns	0.4931
RAD vs. DOX	ns	0.8907
**Collagen Content - Day 7**		
ANOVA summary	3.148	
F	0.0541	
P value	ns	
P value summary	No	
Significant diff. among means (P < 0.05)?	0.3711	
R squared		
**Collagen Content - Day 14**		
ANOVA summary		
F	0.6138	
P value	0.6159	
P value summary	ns	
Significant diff. among means (P < 0.05)?	No	
R squared	0.1032	
**GAG Content - Day 7**		
ANOVA summary		
F	2.236	
P value	0.1235	
P value summary	ns	
Significant diff. among means (P < 0.05)?	No	
R squared	0.2954	
**GAG Content - Day 14**		
Tukey's multiple comparisons test	Summary	Adjusted P Value
**Young vs. Aged**	*******	**0.0004**

**Young vs. RAD**	****	**<0.0001**
**Young vs. DOX**	***	**0.0004**
Aged vs. RAD	ns	0.6187
Aged vs. DOX	ns	>0.9999
RAD vs. DOX	ns	0.6249

Supporting Table S6. Figure 6 statistics. One-way ANOVA at each timepoint with Tukey's multiple comparisons for all groups. ANOVA was significant if multiple comparisons are shown. Day 0 comparisons not shown.

**MMP-1 Gene Expression - Day 7**		
Tukey's multiple comparisons test	Summary	Adjusted P Value
**Young vs. Aged**	**ns**	**0.9757**
**Young vs. RAD**	**ns**	**0.3285**
**Young vs. DOX**	*	**0.0245**
Aged vs. RAD	ns	0.684
Aged vs. DOX	ns	0.115
RAD vs. DOX	ns	0.4508
**MMP-1 Gene Expression - Day 14**		
Tukey's multiple comparisons test	Summary	Adjusted P Value
**Young vs. Aged**	****	**<0.0001**
**Young vs. RAD**	*	**0.0201**
**Young vs. DOX**	***	**0.0007**
Aged vs. RAD	*	0.0151
Aged vs. DOX	ns	0.1891
RAD vs. DOX	ns	0.3162
**MMP-3 Gene Expression - Day 7**		
Tukey's multiple comparisons test	Summary	Adjusted P Value
**Young vs. Aged**	**	**0.0014**
**Young vs. RAD**	*	**0.0211**
**Young vs. DOX**	**Trend**	**0.0643**
Aged vs. RAD	****	<0.0001
Aged vs. DOX	****	<0.0001
RAD vs. DOX	ns	0.9391
**MMP-3 Gene Expression - Day 14**		
Tukey's multiple comparisons test	Summary	Adjusted P Value
**Young vs. Aged**	****	**<0.0001**
**Young vs. RAD**	*	**0.0482**
**Young vs. DOX**	**ns**	**0.4747**
Aged vs. RAD	****	<0.0001
Aged vs. DOX	****	<0.0001
RAD vs. DOX	ns	0.5139

**MMP-13 Gene Expression - Day 7**		
Tukey's multiple comparisons test	Summary	Adjusted P Value
**Young vs. Aged**	********	**<0.0001**
**Young vs. RAD**	**ns**	**0.1708**
**Young vs. DOX**	*****	**0.0325**
Aged vs. RAD	****	<0.0001
Aged vs. DOX	***	0.0003
RAD vs. DOX	ns	0.8038
**MMP-13 Gene Expression - Day 14**		
Tukey's multiple comparisons test	Summary	Adjusted P Value
**Young vs. Aged**	********	**<0.0001**
**Young vs. RAD**	**ns**	**0.9974**
**Young vs. DOX**	**ns**	**0.158**
Aged vs. RAD	****	<0.0001
Aged vs. DOX	****	<0.0001
RAD vs. DOX	ns	0.2167
**MMP Activity- Day 7**		
ANOVA summary		
F	0.209	
P value	0.8882	
P value summary	ns	
Significant diff. among means (P < 0.05)?	No	
R squared	0.04966	
**MMP Activity- Day 12**		
Tukey's multiple comparisons test	Summary	Adjusted P Value
**Young vs. Aged**	**ns**	**0.2717**
**Young vs. RAD**	**ns**	**0.9992**
**Young vs. DOX**	**ns**	**0.472**
Aged vs. RAD	ns	0.3001
Aged vs. DOX	*	0.0218
RAD vs. DOX	ns	0.3707

Supporting Table S7. Figure S1 statistics. One-way ANOVA at each timepoint with Tukey's multiple comparisons for all groups. ANOVA was significant if multiple comparisons are shown.

**Viability - Day 7**		
Tukey's multiple comparisons test	Summary	Adjusted P Value
**Young vs. Aged**	**ns**	**0.948**
**Young vs. RAD**	**Trend**	**0.0588**
**Young vs. DOX**	**ns**	**0.9672**
Aged vs. RAD	ns	0.2861
Aged vs. DOX	ns	0.8371

RAD vs. DOX	ns	0.0667
**Viability - Day 14**		
ANOVA summary		
F	2.222	
P value	0.1343	
P value summary	ns	
Significant diff. among means (P < 0.05)?	No	
R squared	0.3389	
**Cell Density - Day 7**		
ANOVA summary		
F	1.706	
P value	0.2014	
P value summary	ns	
Significant diff. among means (P < 0.05)?	No	
R squared	0.2214	
**Cell Density- Day 14**		
ANOVA summary		
F	3.047	
P value	0.0667	
P value summary	ns	
Significant diff. among means (P < 0.05)?	No	
R squared	0.4128	

Supporting Table S8. Figure S2 statistics. Unpaired t-test between D0 young and 10Gy RAD+ 2hr.

**yH2AX Positive Control**		
Unpaired t test		
P value	<0.0001	
P value summary	****	
Significantly different (P < 0.05)?	Yes	
One- or two-tailed P value?	Two-tailed	

Supporting Table S8. Figure S3 statistics. Unpaired t-test between young and aged groups.

**p16 Gene Expression- Day 0**		
Unpaired t test		
P value	0.0005	
P value summary	***	
Significantly different (P < 0.05)?	Yes	
One- or two-tailed P value?	Two-tailed	
**p53 Gene Expression- Day 0**		
Unpaired t test		

P value	0.0303	
P value summary	*	
Significantly different (P < 0.05)?	Yes	
One- or two-tailed P value?	Two-tailed	
**p21 Gene Expression- Day 0**		
Unpaired t test		
P value	0.0767	
P value summary	Trend	
Significantly different (P < 0.05)?	No	
One- or two-tailed P value?	Two-tailed	
**Lmb1 Gene Expression- Day 0**		
Unpaired t test		
P value	0.2704	
P value summary	ns	
Significantly different (P < 0.05)?	No	
One- or two-tailed P value?	Two-tailed	
**Casp3 Gene Expression- Day 0**		
Unpaired t test		
P value	0.5534	
P value summary	ns	
Significantly different (P < 0.05)?	No	
One- or two-tailed P value?	Two-tailed	
**IL-6 Gene Expression-Day 0**		
Unpaired t test		
P value	0.0086	
P value summary	**	
Significantly different (P < 0.05)?	Yes	
One- or two-tailed P value?	Two-tailed	
**CXCL1 Gene Expression-Day 0**		
Unpaired t test		
P value	0.5087	
P value summary	ns	
Significantly different (P < 0.05)?	No	
One- or two-tailed P value?	Two-tailed	
**MMP-1 Gene Expression- Day 0**		
Unpaired t test		
P value	0.0145	
P value summary	*	
Significantly different (P < 0.05)?	Yes	
One- or two-tailed P value?	Two-tailed	
**MMP-3 Gene Expression-Day 0**		

Unpaired t test		
P value	0.0039	
P value summary	**	
Significantly different (P < 0.05)?	Yes	
One- or two-tailed P value?	Two-tailed	
**MMP-13 Gene Expression- Day 0**		
Unpaired t test		
P value	0.1465	
P value summary	ns	
Significantly different (P < 0.05)?	No	
One- or two-tailed P value?	Two-tailed	
**Col1 Gene Expression- Day 0**		
Unpaired t test		
P value	0.0022	
P value summary	**	
Significantly different (P < 0.05)?	Yes	
One- or two-tailed P value?	Two-tailed	
**FMOD Gene Expression-Day 0**		
Unpaired t test		
P value	0.0415	
P value summary	*	
Significantly different (P < 0.05)?	Yes	
One- or two-tailed P value?	Two-tailed	
**DCN Gene Expression-Day 0**		
Unpaired t test		
P value	0.0354	
P value summary	*	
Significantly different (P < 0.05)?	Yes	
One- or two-tailed P value?	Two-tailed	

Supporting Table S10. Figure S4 statistics. One-way ANOVA at each timepoint with Tukey's multiple comparisons for all groups. ANOVA was significant if multiple comparisons are shown.

**MMP Activity- Day 2**		
ANOVA summary		
F	1.911	
P value	0.1817	
P value summary	ns	
Significant diff. among means (P < 0.05)?	No	
R squared	0.3233	
**MMP Activity- Day 7**		
ANOVA summary		

F	0.209	
P value	0.8882	
P value summary	ns	
Significant diff. among means (P < 0.05)?	No	
R squared	0.04966	
**MMP Activity- Day 12**		
Tukey's multiple comparisons test	Summary	Adjusted P Value
**Young vs. Aged**	**ns**	**0.2717**
**Young vs. RAD**	**ns**	**0.9992**
**Young vs. DOX**	**ns**	**0.472**
Aged vs. RAD	ns	0.3001
Aged vs. DOX	*	0.0218
RAD vs. DOX	ns	0.3707
**IL-6 Concentration- Day 2**		
ANOVA summary		
F	2.149	
P value	0.134	
P value summary	ns	
Significant diff. among means (P < 0.05)?	No	
R squared	0.2872	
**IL-6 Concentration- Day 7**		
ANOVA summary		
F	0.7514	
P value	0.5423	
P value summary	ns	
Significant diff. among means (P < 0.05)?	No	
R squared	0.1581	
**IL-6 Concentration- Day 12**		
Tukey's multiple comparisons test	Summary	Adjusted P Value
**Young vs. Aged**	**ns**	**0.6276**
**Young vs. RAD**	**ns**	**0.3913**
**Young vs. DOX**	**ns**	**0.7379**
Aged vs. RAD	Trend	0.0706
Aged vs. DOX	ns	0.9951
RAD vs. DOX	ns	0.0832
**MIP-1α Concentration- Day 2**		
ANOVA summary		
F	1.778	
P value	0.1918	
P value summary	ns	
Significant diff. among means (P < 0.05)?	No	

R squared	0.2501	
**MIP-1α Concentration- Day 7**		
ANOVA summary		
F	0.09979	
P value	0.959	
P value summary	ns	
Significant diff. among means (P < 0.05)?	No	
R squared	0.01837	
**MIP-1α Concentration- Day 12**		
ANOVA summary		
F	1.939	
P value	0.1528	
P value summary	ns	
Significant diff. among means (P < 0.05)?	No	
R squared	0.2091	
**MCP-1 Concentration- Day 2**		
ANOVA summary		
F	1.428	
P value	0.2715	
P value summary	ns	
Significant diff. among means (P < 0.05)?	No	
R squared	0.2112	
**MCP-1 Concentration- Day 7**		
ANOVA summary		
F	0.8143	
P value	0.5103	
P value summary	ns	
Significant diff. among means (P < 0.05)?	No	
R squared	0.1691	
**MCP-1 Concentration- Day 12**		
Tukey's multiple comparisons test	Summary	Adjusted P Value
**Young vs. Aged**	**ns**	**0.3288**
**Young vs. RAD**	*****	**0.0343**
**Young vs. DOX**	**Trend**	**0.0913**
Aged vs. RAD	ns	0.9393
Aged vs. DOX	ns	0.958
RAD vs. DOX	ns	>0.9999
**IL-13 Concentration- Day 2**		
ANOVA summary		
F	0.2553	
P value	0.8557	

P value summary	ns	
Significant diff. among means (P < 0.05)?	No	
R squared	0.07842	
**IL-13 Concentration- Day 7**		
Incomplete data set		
**IL-13 Concentration- Day 12**		
ANOVA summary		
F	0.491	
P value	0.6925	
P value summary	ns	
Significant diff. among means (P < 0.05)?	No	
R squared	0.0686	
**TNF-α Concentration- Day 2**		
ANOVA summary		
F	1.959	
P value	0.1608	
P value summary	ns	
Significant diff. among means (P < 0.05)?	No	
R squared	0.2687	
**TNF-α Concentration- Day 7**		
ANOVA summary		
F	0.4163	
P value	0.7445	
P value summary	ns	
Significant diff. among means (P < 0.05)?	No	
R squared	0.09426	
**TNF-α Concentration- Day 12**		
ANOVA summary		
F	1.471	
P value	0.2527	
P value summary	ns	
Significant diff. among means (P < 0.05)?	No	
R squared	0.1807	
**KC-GRO Concentration- Day 2**		
ANOVA summary		
F	2.255	
P value	0.1212	
P value summary	ns	
Significant diff. among means (P < 0.05)?	No	
R squared	0.2972	
**KC-GRO Concentration- Day 7**		

ANOVA summary		
F	1.751	
P value	0.21	
P value summary	ns	
Significant diff. among means (P < 0.05)?	No	
R squared	0.3044	
**KC-GRO Concentration- Day 12**		
ANOVA summary		
F	2.621	
P value	0.0775	
P value summary	ns	
Significant diff. among means (P < 0.05)?	No	
R squared	0.2724	
**GM-CSF Concentration- Day 2**		
ANOVA summary		
F	3.237	
P value	0.0501	
P value summary	ns	
Significant diff. among means (P < 0.05)?	No	
R squared	0.3777	
**GM-CSF Concentration- Day 7**		
Incomplete data set		
**GM-CSF Concentration- Day 12**		
Incomplete data set		

Supporting Table S11. Figure S5 statistics. Unpaired t-test between Young and DOX groups at day 7.

**Metabolic Activity Day 7**		
Unpaired t test		
P value	0.0516	
P value summary	Trend	
Significantly different (P < 0.05)?	No	
One- or two-tailed P value?	Two-tailed	
**DNA Content Day 7**		
Unpaired t test		
P value	0.197	
P value summary	ns	
Significantly different (P < 0.05)?	No	
One- or two-tailed P value?	Two-tailed	
**Proliferation Day 7**		
Unpaired t test		

P value	<0.0001	
P value summary	****	
Significantly different (P < 0.05)?	Yes	
One- or two-tailed P value?	Two-tailed	
**p16 Gene Expression- Day 7**		
Unpaired t test		
P value	0.0402	
P value summary	*	
Significantly different (P < 0.05)?	Yes	
One- or two-tailed P value?	Two-tailed	
**p53 Gene Expression- Day 7**		
Unpaired t test		
P value	0.0013	
P value summary	**	
Significantly different (P < 0.05)?	Yes	
One- or two-tailed P value?	Two-tailed	
**p21 Gene Expression- Day 7**		
Unpaired t test		
P value	<0.0001	
P value summary	****	
Significantly different (P < 0.05)?	Yes	
One- or two-tailed P value?	Two-tailed	
**Lmb1 Gene Expression- Day 7**		
Unpaired t test		
P value	0.0883	
P value summary	Trend	
Significantly different (P < 0.05)?	No	
One- or two-tailed P value?	Two-tailed	
**Casp3 Gene Expression- Day 7**		
Unpaired t test		
P value	0.0337	
P value summary	*	
Significantly different (P < 0.05)?	Yes	
One- or two-tailed P value?	Two-tailed	
**IL-6 Gene Expression- Day 7**		
Unpaired t test		
P value	0.0093	
P value summary	**	
Significantly different (P < 0.05)?	Yes	
One- or two-tailed P value?	Two-tailed	
**Collagen 1 Gene Expression- Day 7**		

Unpaired t test		
P value	0.0019	
P value summary	**	
Significantly different (P < 0.05)?	Yes	
One- or two-tailed P value?	Two-tailed	
**DCN Gene Expression- Day 7**		
Unpaired t test		
P value	0.022	
P value summary	*	
Significantly different (P < 0.05)?	Yes	
One- or two-tailed P value?	Two-tailed	
**FMOD Gene Expression- Day 7**		
Unpaired t test		
P value	0.0168	
P value summary	*	
Significantly different (P < 0.05)?	Yes	
One- or two-tailed P value?	Two-tailed	
**MMP-1 Gene Expression- Day 7**		
Unpaired t test		
P value	0.3786	
P value summary	ns	
Significantly different (P < 0.05)?	No	
One- or two-tailed P value?	Two-tailed	
**MMP-3 Gene Expression- Day 7**		
Unpaired t test		
P value	0.0778	
P value summary	Trend	
Significantly different (P < 0.05)?	No	
One- or two-tailed P value?	Two-tailed	
**MMP-13 Gene Expression- Day 7**		
Unpaired t test		
P value	0.5463	
P value summary	ns	
Significantly different (P < 0.05)?	No	
One- or two-tailed P value?	Two-tailed	
**MMP Activity- Day 7**		
Unpaired t test		
P value	0.7737	
P value summary	ns	
Significantly different (P < 0.05)?	No	
One- or two-tailed P value?	Two-tailed	

**IL-6 Concentration- Day 7**		
Unpaired t test		
P value	0.4677	
P value summary	ns	
Significantly different (P < 0.05)?	No	
One- or two-tailed P value?	Two-tailed	
**MIP-1α Concentration- Day 7**		
Unpaired t test		
P value	0.4151	
P value summary	ns	
Significantly different (P < 0.05)?	No	
One- or two-tailed P value?	Two-tailed	
**MCP-1 Concentration- Day 7**		
Unpaired t test		
P value	0.266	
P value summary	ns	
Significantly different (P < 0.05)?	No	
One- or two-tailed P value?	Two-tailed	
**TNF-α Concentration- Day 7**		
Unpaired t test		
P value	0.5438	
P value summary	ns	
Significantly different (P < 0.05)?	No	
One- or two-tailed P value?	Two-tailed	
**KC-GRO Concentration- Day 7**		
Unpaired t test		
P value	0.5263	
P value summary	ns	
Significantly different (P < 0.05)?	No	
One- or two-tailed P value?	Two-tailed	
